# Renal clearance of graphene oxide: glomerular filtration or tubular secretion and selective kidney injury association with its lateral dimension

**DOI:** 10.1186/s12951-023-01781-x

**Published:** 2023-02-10

**Authors:** Wei Chen, Bing Wang, Shanshan Liang, Meng Wang, Lingna Zheng, Si Xu, Jiali Wang, Hao Fang, Pu Yang, Weiyue Feng

**Affiliations:** 1 CAS Key Laboratory for Biomedical Effects of Nanomaterials and Nanosafety, Institute of High Energy Physics, Chinese Academy of Sciences, Beijing, 100049 China; 2 University of Chinese Academy of Sciences, Beijing, 100049 China; 3 School of Pharmacy, Yantai University, Yantai, 264005 China

**Keywords:** Glomerular filtration, Tubular secretion, Graphene oxide, Renal excretion mechanism, Selective kidney injury, Lateral dimension

## Abstract

**Background:**

Renal excretion is one of the major routes of nanomaterial elimination from the body. Many previous studies have found that graphene oxide nanosheets are excreted in bulk through the kidneys. However, how the lateral size affects GO disposition in the kidneys including glomerular filtration, active tubular secretion and tubular reabsorption is still unknown.

**Results:**

The thin, two-dimensional graphene oxide nanosheets (GOs) was observed to excrete in urine through the kidneys, but the lateral dimension of GOs affects their renal clearance pathway and renal injury. The *s*-GOs could be renal excreted via the glomerular filtration, while the *l*-GOs were predominately excreted via proximal tubular secretion at a much faster renal clearance rate than the s-GOs. For the tubular secretion of *l*-GOs, the mRNA level of basolateral organic anion transporters *Oat1* and *Oat2* in the kidney presented dose dependent increase, while no obvious alterations of the efflux transporters such as *Mdr1* and *Mrp4* mRNA expression levels were observed, suggesting the accumulation of *l*-GOs. During the GO renal elimination, mostly the high dose of 15 mg/kg *s*-GO and *l*-GO treatment showed obvious kidney injuries but at different renal compartment, i.e., the *s*-GOs induced obvious glomerular changes in podocytes, while the *l*-GOs induced more obvious tubular injuries including necrosis of renal tubular epithelial cells, loss of brush border, cast formation and tubular dilatation. The specifically tubular injury biomarkers KIM1 and NGAL were shown slight increase with mRNA levels in *l*-GO administrated mice.

**Conclusions:**

This study shows that the lateral size of GOs affected their interactions with different renal compartments, renal excretion pathways and potential kidney injuries.

**Supplementary Information:**

The online version contains supplementary material available at 10.1186/s12951-023-01781-x.

## Background

Over the past few decades, many nanomedicines play a pivotal role in the diagnosis, imaging and treatment of life-threatening diseases including cancer, but concerns about the toxic effects of nanomaterials (NMs) still linger [1]. The kidney is a vital organ for blood filtration, waste elimination and essential substance reabsorption and, importantly, responsible for the elimination of a myriad of drugs and the endogenous compounds. Therefore, evaluating and improving kidney safety is an active research topic and a requirement in the early stage of drug development. As for nanomedicine, the kidneys undergo the key roles in the transport and clearance of NMs in vivo thus become one of the most vulnerable organs after NM prolonged exposure [2–4]. Meanwhile, renal-excretable nanomedicines are expectedly to greatly reduce toxicity for the benefit of successfully clinical translation. For this reason, understanding NM-kidney interactions are crucially required to reveal the underlying principles of NM accumulation, targeting, clearance and toxicity in the kidney.

It is known that the renal clearance of drugs is the net results of glomerular filtration plus tubular secretion minus tubular reabsorption. Thus, once NMs enter into the renal system with blood flow, the NM-kidney interactions may occur at glomerulus and renal tubules [3, 5]. Multiple evidences have shown that the overall cut-off size of 6–8 nm of NMs are thought to be excreted by crossing the glomerular filtration barrier (GFB) [6, 7]. However, so far, NMs are seldom observed involving in the tubular secretion and reabsorption due to the weak interaction of NMs with renal tubular cells. Some recent studies have shown that though some NMs with diameters larger than 100 nm, they could be secreted by proximal tubular epithelial cells, subsequently through the tubular system excretion into urine [8–10]. For instance, 130–180 nm sized spherical poly lactic-co-glycolic acid (PLGA) polymeric nanoparticles (NPs) were found to undergo rapid renal clearance via proximal tubule secretion [8]. The large “mesoscale” (250–400 nm) NPs were selectively targeted the proximal tubules via tubular secretion pathway where the NPs were endocytosed by proximal tubule epithelial cells via transcytosis of peritubular capillary endothelial cells [9–11]. The enhanced tubular reabsorption was once observed in the injured mouse kidneys for the renal clearable gold nanoparticles [7].

Graphene oxide (GO) NMs have been widely used for drug delivery, biological sensor, photodynamic therapy, cancer therapy, antibacterial therapy and so forth due to its unique physicochemical properties, such as planar structure, high surface area, strong mechanical strength, good biocompatibility, etc. [12–17]. Several recent researches have shown that GO nanosheet treatment could enhance inhibition of SARS-CoV-2 and increase immune responses against the virus [18, 19]. Excitingly, many previous studies including ours have shown that GO nanosheets (GOs) could be cleared by the kidneys into urine after intravenous (i.v.) injection [20–23]. The previous researches revealed that the thin, well-dispersed, and flexible GOs could pass through the GFB by rolling, crumpling or folding of the nanosheets and then excreted in the urine, while no obvious impairment of kidney function was found [21, 22]. However, the affection of differential lateral sizes of GOs on the renal excretion pathways has not known yet, especially, whether the proximal tubular secretion and reabsorption involves in renal elimination of GOs is seldom known. Moreover, the lateral size of GOs has been accepted as an important parameter to regulate cellular uptake and biological responses [24–29], but how the lateral dimension of GOs influence GO interactions with different renal compartments is still unclear.

Herein, we prepared GOs with two lateral sizes, the small sized GOs (*s*-GOs) and the large sized GOs (*l*-GOs). We investigated the effects of lateral size of GOs on the renal excretion pathways after intravenous injection of *s*-GOs and *l*-GOs in mice. Using several in vivo and in vitro imaging techniques, we revealed that both the *s*-GOs and *l*-GOs could be eliminated by the kidneys in the urine, whereas, we found the renal excretion of *l*-GOs was predominately via proximal tubular secretion and its renal clearance rate was much faster than that of *s*-GOs. We explored the mechanism of the tubular secretion of GOs and identified that several renal drug transporters that expressed in the basolateral and optical membrane of renal proximal tubules had involved in the uptake and efflux of GOs in the renal tubule. Assessment of kidney injury indicates the dose-dependent histopathological changes in glomerulus and renal tubule during the GO renal elimination. We further detected several biomarkers as the early signs of kidney injury, including the level of albuminuria, the mRNA expression levels of kidney injury molecule-1 (KIM1) and neutrophil gelatinase-associated lipocalin (NGAL). Our findings provide new insight into the processes of renal excretion of GOs, demonstrating the influence of lateral dimension of GO, therefore provide a chance of tuning lateral size of GOs for biomedical application with reduced renal toxicity.

## Methods

### Chemicals and materials

High quality GOs (purity > 99%) was purchased from Jiangsu XFNANO materials Tech. Co. Ltd., China. 1-Ethyl-3-(3-dimethylaminopropyl) carbodiimide (EDC) and N-hydroxysuccinimide (NHS) were obtained from Shanghai Aladdin Biochemical Tech. Co. Ltd., China. 2-(4-isothiocyanatobenzyl)-1,4,7,10 tetraazacyclododecane-1,4,7,10-tetraacetic acid (DOTA-NCS) was from Macrocyclics (Dallas, TX, USA). Ytterbium nitrate (Yb(NO_3_)_3_⋅5H_2_O, 99.9%) was obtained from Strem Chemicals, Inc., USA. Aminated PEG (molecular weight ~ 5 kDa) and indocyanine green (ICG) were purchased from Beijing J&K Scientific Ltd. Fluorescein isothiocyanate (FITC) was obtained from Beijing Liangdong Technology Co. Ltd.

### PEGylated s-GOs and l-GOs preparation and rare earth element Yb labeling

The small lateral sized *s*-GOs and large lateral sized *l*-GOs were prepared via ultrasonication exfoliation. The PEGylated GOs was synthesized according to our previous study [30]. Briefly, *s*-GOs and *l*-GOs were separately prepared via ultrasonic exfoliation in an ice-water bath for 10 h and 10 min, respectively. After that, PEGylated GOs were prepared via EDC/NHS coupling chemistry at room temperature. The obtained PEGylated *s*-GOs and *l*-GOs were purified by centrifugation at 14,000 rpm and 6000 rpm for 10 min, respectively, and then dialyzed for 12 h. The labeling of rare earth element (REE) Yb on GOs was performed via DOTA linkage (Additional file 1: Fig. S1). The labeling efficiency of Yb on GO was obtained by calculating the ratio of the remained amount of Yb on *s*-GOs/*l*-GOs to the total amount of mass on the complex that was determined with xylenol orange [30].

### Fluorescence labeled s-GOs and l-GOs preparation

Indocyanine green (ICG-NHS)-labeled *s*-GOs (*s*-GOs/ICG) and *l*-GOs (*l*-GOs/ICG) were prepared by an amide reaction between the ICG-NHS and PEG-amine on the surface of GOs. In short, 0.5 mg/mL GOs was added into 0.1 mg/mL ICG-NHS in DMSO solvent, followed by stirring for 3 h in the dark at 4℃.The mixture was centrifuged at 6000 rpm via ultrafiltration tube with a molecular weight cutoff of 10 kDa for several times to remove the unreacted free ICG. The concentration of GOs/ICG was determined by fluorescence spectroscopy at 820 nm.

FITC-labeled GOs were prepared by diimide-activated amidation between FITC-BSA and amine of PEGylated GO via EDC/NHS activation. In brief, GO suspension was mixed with 5 mg EDC and 10 mg NHS and stirred for 1 h at room temperature. The precipitate was collected by centrifugation at 18,500*g* for 0.5 h, then resuspended in sterilized water and reacted with 0.1 mg/mL FITC-BSA solution in the dark for 24 h. The collected precipitate was centrifuged at 18,500*g* for several times to remove the unreacted FITC-BSA. The final precipitate was suspended in 1 mL sterile water and stored at 4 °C for further use.

### Physicochemical characterization of *s*-GOs and *l*-GOs

The thickness, size and morphology of the *s*-GOs and *l*-GOs were characterized by atomic force microscopy (AFM, AFM5500, Bruker, UK) and transmission electron microscopy (TEM, JEOL, JEM-2100F). The content of functional groups on GO was analyzed by means of synchrotron radiation X-ray photoelectron spectroscopy (SR-XPS) at 4B9B beamline of Beijing Synchrotron Radiation Facility (BSRF), China. Raman spectra were measured using a WITec Alpha 300 R Raman spectrometer (WITec, Germany) with a 532 nm laser. The concentration of Yb on GOs was determined by inductively coupled plasma mass spectrometry (ICP-MS, NexION 300D, PerkinElmer, Inc., USA).

### Animals and treatments

All animal experiments were approved by the Institutional Animal Care and Use Committee. The male CD-1 (ICR) mice (7-week old, body weight ~ 20 g) were purchased from Beijing Vital River Laboratory Experimental Animal Technology Co. Ltd. (Beijing, China). All mice were housed 6 per cage in an air-conditioned room (22 ± 2 °C, relative humidity 45 ± 5%) under a regular 12-h light/dark cycle and fed with standard chow and deionized water ad libitum. Following one week of adaptation, *s*-GOs and *l*-GOs were intravenously (i.v.) injected into the mice through the tail vein at doses of 1, 5 and 15 mg/kg body weight (bw), of which the dose is comparable to the amount commonly used in biomedical applications. For the control groups, 200 μL of saline solution was i.v. injected instead.

The serum samples were collected at day 7 and 28 post-injection for biochemical assays. The biochemical markers of renal function including creatinine (CREA), blood urine nitrogen (BUN) and uric acid (UA) levels were analyzed. The biochemical markers of liver function: alanine aminotransferase (ALT), aspartate aminotransferase (AST), and alkaline phosphatase (ALP) were also analyzed. One random of the kidney of the control and injected mice were harvested at day 7 and 28 post-injection and fixed in 4% paraformaldehyde for histopathological examination using hematoxylin–eosin (H&E) staining. The 24-h urine samples were collected at 24–48 h and day 6–7 after i.v. injection for the measurement of albumin to creatinine ratio (ACR). The details are provided in supplementary material.

### Confocal Raman spectroscopy analysis

Confocal Raman imaging of GOs in the kidney sections was performed using a WITec alpha 300 R confocal Raman microscope (WITec, Germany) with a 532 nm laser excitation source. The Raman signal was acquired by a Peltier-cooled CCD (− 70 °C) detector using a 600 line/mm grating spectrograph (UHTS 300, WITec, Ulm, Germany). Raman images were obtained at a 500 ms/point integration time in a 0.5 × 0.5 µm^2^ pixel resolution.

### LA-ICP-MS elemental imaging analysis

In order to visualize the micro-distribution of *s*-GOs and *l*-GOs in the kidney, the Yb-labelled GOs (*s*-GOs/Yb and *l*-GOs/Yb) was imaged by a laser ablation (NWR213 laser ablation system, Elemental Scientific Lasers, Bozeman, USA) coupled ICP-MS system (LA-ICP-MS). Before imaging analysis, the kidney samples were perfused with saline solution followed by continued fixation with 4% paraformaldehyde. The collected samples were frozen and sliced into 10 μm thick slices. LA-ICP-MS elemental imaging was conducted at a 60 μm spot size and a scanning speed of 60 μm/sec. The obtained data were analyzed by Igor Pro 6.0 software (Wavemetrics, Lake Oswego, OR, USA).

### In vivo fluorescence imaging

BALB/c nude mice (Female, body weight ~ 20 g) were obtained from Beijing Vital River Laboratory Experimental Animal Technology Co. Ltd. (Beijing, China). To visualize the distribution and transport patterns of *s*-GOs and *l*-GOs in vivo, 200 µL (200 µg/mL of GOs) ICG-NHS labelled *s*-GOs (*s*-GOs/ICG) and *l*-GOs (*l*-GOs/ICG) were i.v. injected into the mice (n = 3). The mice were anesthetized with isoflurane, then the real time near-infrared fluorescence imaging was performed on an IVIS Spectrum System (Perkin Elmer, USA) using 745 nm excitation and 820 nm emission filter. The ex vivo fluorescence imaging for the intact tissues, including the liver, spleen, lung and kidneys, was also performed at 1, 4, 24, 48 h and 7 d after injection of *s*-GOs/ICG and *l*-GOs/ICG into the mice. The mice treated with saline and free ICG were used as the negative and positive controls.

### TEM imaging

The ultrastructural changes of the kidney after GOs treatment were observed by TEM (JEOL JEM-1400) imaging. The kidney tissues were prefixed with 2.5% glutaraldehyde at 4 °C overnight and then post-fixed in 1% osmium tetroxide at 4 °C for 3 h. The samples were sectioned to 70 nm thick after dehydration and resin embedding, stained with uranyl acetate and lead citrate. The glomerular ultrastructural changes, including the glomerular basement membrane (GBM) thickness, podocyte foot process width and length, and glomerular slit diaphragm perimeter were quantitatively analyzed using ImageJ 1.51 k software.

### Analysis of cellular uptake, exocytosis, and cell viability of GOs in HK-2 cells

The human renal tubular epithelial cell line (HK-2) was obtained from Procell (Wuhan, China). The uptake and intracellular fate of *s*-GOs and *l*-GOs in HK-2 cells was visualized and quantified by confocal fluorescence microscopy (CFM, LU-N4 Laser Unit, Nikon) to evaluate the renal tubular uptake and exocytosis of the GOs. HK-2 cells were incubated with 10 μg/mL of FITC labelled *s*-GOs or *l*-GOs for 4 h and 24 h. The cellular nuclei and lysosome were stained with DAPI and LysoTracker Red, respectively. For exocytosis analysis of *s*-GOs or *l*-GOs from HK-2 cells, after 24 h exposure, the cell culture medium was removed then the HK-2 cells were washed thoroughly with fresh GO-free medium and continuously cultured with fresh medium for another 24 h. The HK-2 cells were washed for three times with PBS and were subsequently stained with DAPI and LysoTracker Red for CFM observation. The exocytosis of *s*-GOs or *l*-GOs were quantitatively analyzed by tracking of intracellular uptake of *s*-GOs or *l*-GOs. The semi-quantitative statistics on fluorescence images of *s*-GOs/FITC and *l*-GOs/FITC in HK-2 cells were performed based on four different areas. Further, flow cytometry analysis was also used to investigate the intracellular uptake of FITC-GO. HK-2 cells were cultured in 6-wells plate and incubated with 10 μg/mL FITC labelled *s*-GO and *l*-GO for 24 h respectively. After washing by PBS for three times, the cells were collected and fixed using 4% paraformaldehyde for 15 min at room temperature. Then the quantitative intracellular uptake of FITC-GO sheets was subsequently analyzed using the percentage of side scatter (SSC). Cell viability was measured using the cell counting kit-8 (CCK-8) assay (Beyotime Biotech., China). HK-2 cells were seeded into 96-wells plates at a density of 1.0 × 10^4^ cells per well, with five duplicate wells in each group. When 80–90% confluence was reached, cells were separately treated with *s*-GOs and *l*-GOs at 1, 10, 25, 50, 100, and 200 μg/mL for 24 h. The relative cell viability was calculated as the percentage of untreated cells.

### RNA extraction and quantitative RT-PCR assay

Total RNA of the frozen kidney tissue samples was extracted with Trizol reagent (Beyotime, China). The extracted RNA was qualitatively measured in NanoDrop One Microvolume UV–Vis Spectrophotometer (ThermoFisher, MA, USA). The extracted RNA was retro transcribed to complementary DNA (cDNA) by using an RNA-to-cDNA kit (Beyotime, China). RT-PCR was performed on a CFX Connect Real-Time PCR Detection system (Bio-Rad) by using the BeyoFast™ SYBR Green qPCR Mix (Beyotime, China). For quantitative results, the mRNA levels are expressed as a fold change by the 2^−ΔΔCt^ method using GAPDH as an endogenous control. The list of primers used in this study is provided in Additional file 1: Table S1.

### Statistical analysis

All of the data were presented as mean ± standard deviation. Student’s t-test or one-way analysis of variance (ANOVA) were used to compare the statistical differences between groups. A value of *p* < 0.05 was considered statistically significant. Significance levels were set at *^, #^*p* < 0.05, **^, ##^*p* < 0.01 and ***^, ###^*p* < 0.001.

## Results

### Physicochemical properties of s-GOs and l-GOs

**Fig. 1:**
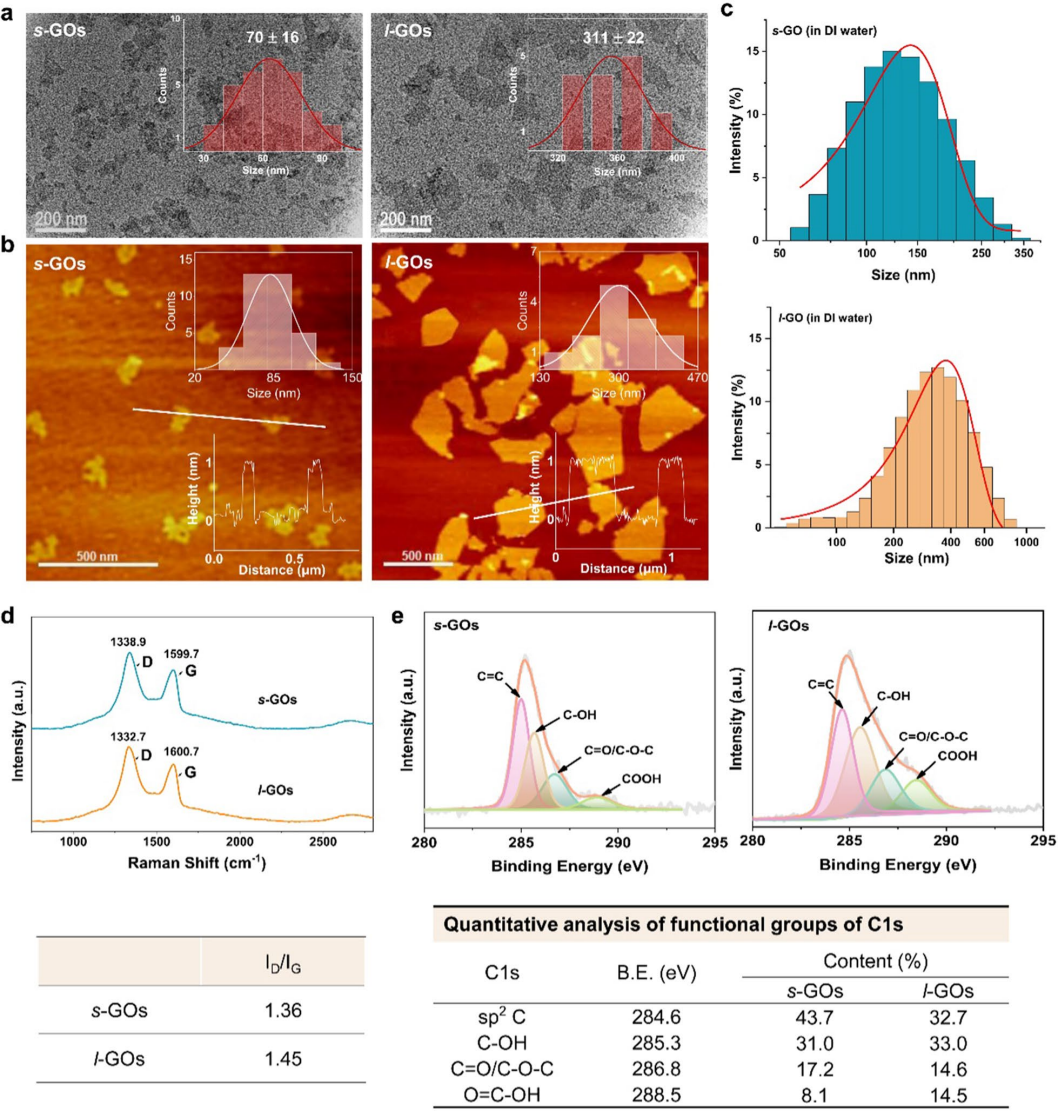
Physicochemical characterization of GO nanosheets (GOs). **a** TEM images of *s*-GOs and *l*-GOs. The insert figures indicate the statistical distribution of lateral size of GOs. **b** AFM images of *s*-GOs and *l*-GOs. The insert figures on the top-right and bottom-right indicate the size and height measurements, respectively. **c** Hydrodynamic size distributions of *s*-GOs and *l*-GOs in deionized water. **d** Raman spectra of *s*-GOs and *l*-GOs. **e** XPS spectra of *s*-GOs and *l*-GOs and quantitative analysis of functional groups of C1s

The representative TEM images of GOs confirmed that both *s*-GOs and *l*-GOs had a typical flake-like structure (Fig. 1a). AFM image analysis shows the average lateral dimension of *s*-GOs and *l*-GOs is 70 and 311 nm, respectively, and the height is 1.02 and 1.05 nm, respectively (Fig. 1b). The dispersibility of GOs in aqueous solutions was assessed by hydrodynamic diameter (HD) and zeta potential measurements in deionized water. The HDs of *s*-GOs and *l-*GOs are 162 and 330 nm (Fig. 1c), respectively, which are slightly larger than the primary size measured by AFM (Table 1). The *s*-GOs and *l*-GOs showed negative zeta potentials at − 10.3 and − 12.9 mV, respectively, indicating that the amine groups in PEG had neutralized some negatively charged carboxylic acid groups in GOs. The Raman spectrum of *s*-GOs consists of a typical D band at 1338.9 cm^−1^ and G band at 1599.7 cm^−1^, as for *l-*GOs, D band at 1332.7 cm^−1^ and G band at 1600.7 cm^−1^. The I_D_/I_G_ ratios in *s*-GO and *l*-GO are calculated as 1.36 and 1.45, respectively, indicating that the GO powders have similar oxidation degree (Fig. 1d). High resolution XPS spectrum of C 1 s (Fig. 1e) show a dominant peak at 284.6 eV, which is assigned to graphitic C = C species, and the other three peaks at 285.7, 286.8, and 288.5 eV are corresponding to C–OH, C–O–C/C = O, and O = C–OH, respectively. Further quantitative analysis indicates that the *s*-GOs and *l*-GOs possess similar surface chemistry. The labeling efficiency of Yb^3+^ on *s*-GOs and *l*-GOs is 35.8 and 42.3 mg/g, respectively (Table 1). Moreover, the Yb^3+^ labelled *s*-GOs and *l*-GOs showed very well dispersion stability in saline solution that more than 99% of Yb was stably labelled on *s*-GOs and *l*-GOs for at least 7 days (Additional file 1: Fig. S2), which is in agreement with our previous report [22].

**Table 1:** Physicochemical properties of s-GOs and *l*-GOs

Sample	Measured by AFM (nm)	HD (nm)	Zeta potential (mV)	Yb on GOs (mg/g)
*s*-GOs	70.1 ± 16.2	162 ± 9	− 10.3 ± 1.1	35.8
*l*-GOs	311.2 ± 21.7	300 ± 24	− 12.9 ± 0.7	42.3

*HD* Hydrodynamic diameter

### s-GOs and l-GOs show renal clearance and urinary excretion

The GOs/ICG was used as fluorescence probe to trace the in vivo transport and clearance of GOs in mice after i.v. injection (Additional file 1: Fig. S3). The in vivo and ex vivo fluorescence detection and imaging clearly indicate that *s*-GOs and *l*-GOs had similar hepatic and renal clearance patterns (Additional file 1: Fig. S3a–c). The obvious fluorescence signals were observed in the abdomen at 10 min post-injection and the kidney signals increased from 0.5 to 1 h and then gradually decreased. At day 7 after injection, only weak fluorescence signals were observed in *s*-GO and *l*-GO treated mice (Additional file 1: Fig. S3c). The fluorescence could be observed in the bladder (Additional file 1: Fig. S3b), suggesting the urinary excretion of GOs. The semiquantitative analysis shows that compared with the fluorescence intensity at 1 h post-injection, about 37% of *s*-GOs and 60% of *l*-GOs were cleared at 4 h post-injection, and about 91% of *s*-GO and 96% of *l*-GO were cleared at 24 h post-injection, indicating efficient renal clearance of both *s*-GOs and *l*-GOs (Additional file 1: Fig. S3d).

**Fig. 2:**
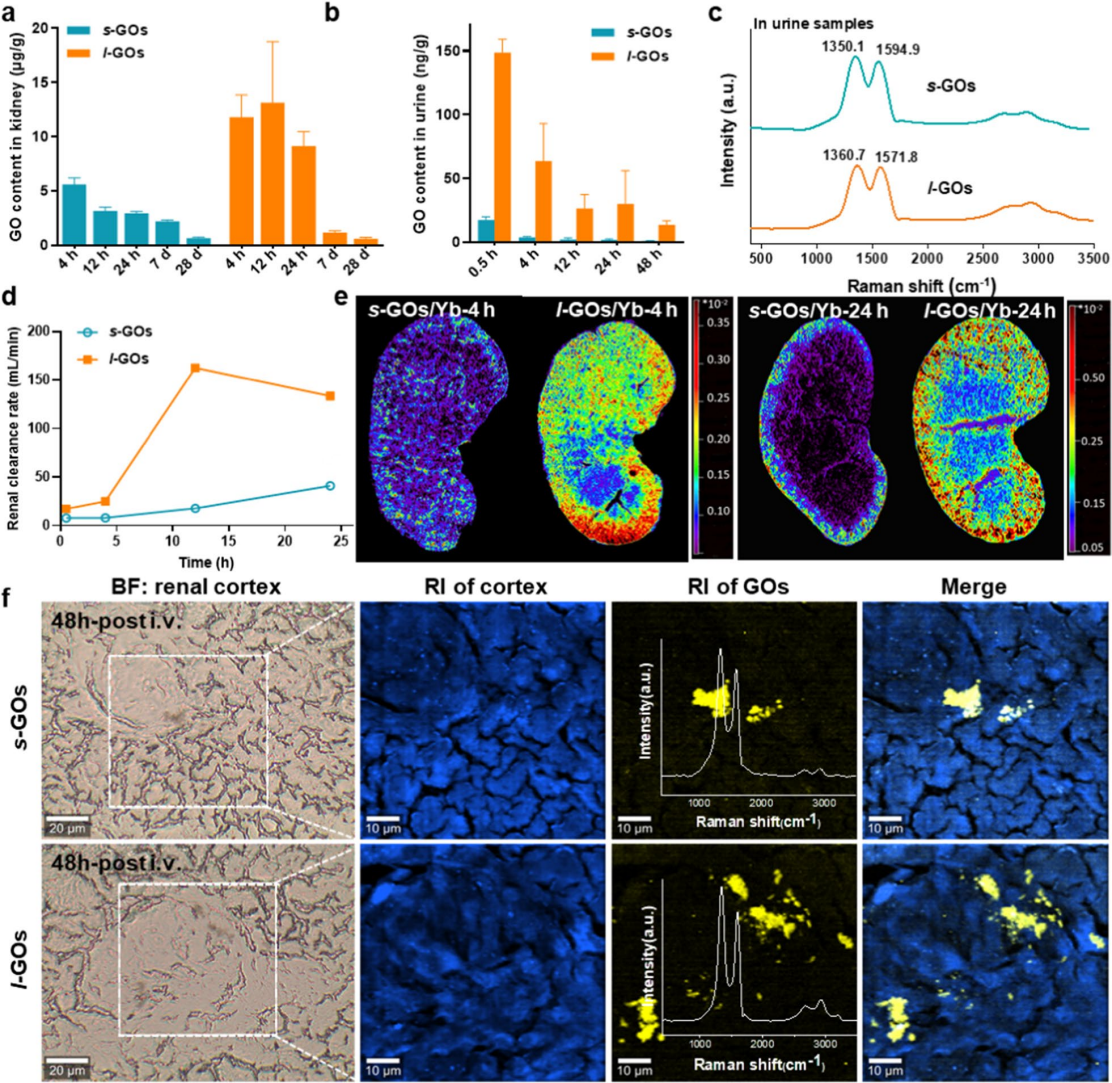
Renal clearance and urinary excretion of *s*-GOs and *l*-GOs after *i.v.* injection in mice. **a** Renal clearance patterns of *s*-GOs and *l*-GOs after i.v injection. **b** Urinary excretion kinetics of *s*-GOs and *l*-GOs. **c** Raman spectra of urine samples of *s*-GO and *l*-GO administrated mice. **d** Renal clearance rate of *s*-GOs and *l*-GOs. Renal clearance rate is the ratio of urinary excretion of GO ([U] × v) to plasma concentration. [U]: urinary GO concentration; v: urine flow rate (0.002 ml/min). **e** LA-ICP-MS images of the distribution of Yb labelled GOs (*s*-GOs/Yb and *l*-GOs/Yb) in the kidney at 4 and 24 h post-injection. **f** Raman spectral images of *s*-GOs and *l*-GOs in renal cortex at 48 h after i.v. injection. From left to right: Bright field (BF) image; Raman image (RI) of autofluorescence (blue color) or GOs (yellow color, obtained from Raman spectrometry); merge image of tissue and GOs

The pharmacokinetics of Yb labelling *s*-GOs and *l*-GOs (*s*-GOs/Yb and *l*-GOs/Yb) was detected by ICP-MS (Additional file 1: Fig. S4, S5). The data show that after 4 h of i.v. injection, the content of GOs in the kidneys of *l*-GO treated mice was two times higher than that in *s*-GO treated mice (Fig. 2a). The renal accumulation of both *s*-GOs and *l*-GOs shows gradual decrease from 4 h to day 28 post-injection, indicating that the GOs had been cleared from the kidneys. At day 28 after injection, the contents of GOs in the kidneys of *s*-GO and *l*-GO treated mice are similar, only 0.6–0.7 μg/g GOs retained (Fig. 2b), which agree with the results of in vivo fluorescence imaging. The Raman signatures of GO had been detected in urine of *s*-GO and *l*-GO group mice, demonstrating the urinary excretion of intact GOs (Fig. 2c). In the urine samples, the D and G bands appeared little wider than the primary GOs, and the I_D_/I_G_ ratios slightly increased to 1.58 (vs 1.36) in *s*-GO and to 1.65 (vs 1.45) in *l*-GO treated mice, suggesting the increase of the structural disorder of GOs during circulation and urinary excretion. Further calculation of the renal clearance rate demonstrates that the renal excretion of GOs in *l*-GO mice was faster than that in *s*-GO treated mice (Fig. 2d).

The LA-ICP-MS images visually display the transfer and clearance pathways of GOs/Yb in the kidney parenchyma (Fig. 2e). The images show that the signal intensity of GOs/Yb in *l*-GO treated mice are obviously higher than that in *s*-GO treated mice. At 4 h after injection, the *l*-GOs were transferred from the renal artery to the renal cortex and renal medulla with the blood flow and primarily distributed in the renal cortex. At 24 h post-injection, the signals of *s*-GOs/Yb and *l*-GOs/Yb in the renal cortex and renal medulla decreased, indicating the renal clearance of GOs.

Further, the accumulation of GOs in kidneys was identified by confocal Raman spectroscopy. The signatures of GO had been detected in the renal cortex of both *s*-GO and *l*-GO treated mice at 48 h after injection, demonstrating GO distributed in the kidneys (Fig. 2f). The I_D_/I_G_ ratios in the kidney of *s*-GO (I_D_/I_G_ ≈ 1.45) and *l*-GO (I_D_/I_G_ ≈ 1.61) treated mouse slightly increased, in accordance with the data of urine samples. Consistent results were also obtained by the FTIR imaging (Additional file 1: Fig. S6).

In addition, the distribution patterns of *s*-GOs and *l*-GOs in other organs show that both the *s*-GOs and *l*-GOs were prone to accumulate in mononuclear phagocyte system (MPS) including the liver, spleen and lung (Additional file 1: Fig. S5). Besides, an amount of GOs stored in the small intestine, which is in accordance with the data of feces excretion in *s*-GO and *l*-GO treated mice (Additional file 1: Fig. S7). Further, compared with the control, there were no significant changes for the functional markers of the liver at 48 h and 7 d post-injection of 15 mg/kg *s*-GOs and *l*-GOs to mice. (Additional file 1: Fig. S8).

### Glomerular filtration and tubular secretion were shown involving in s-GO and l-GO renal elimination

**Fig. 3:**
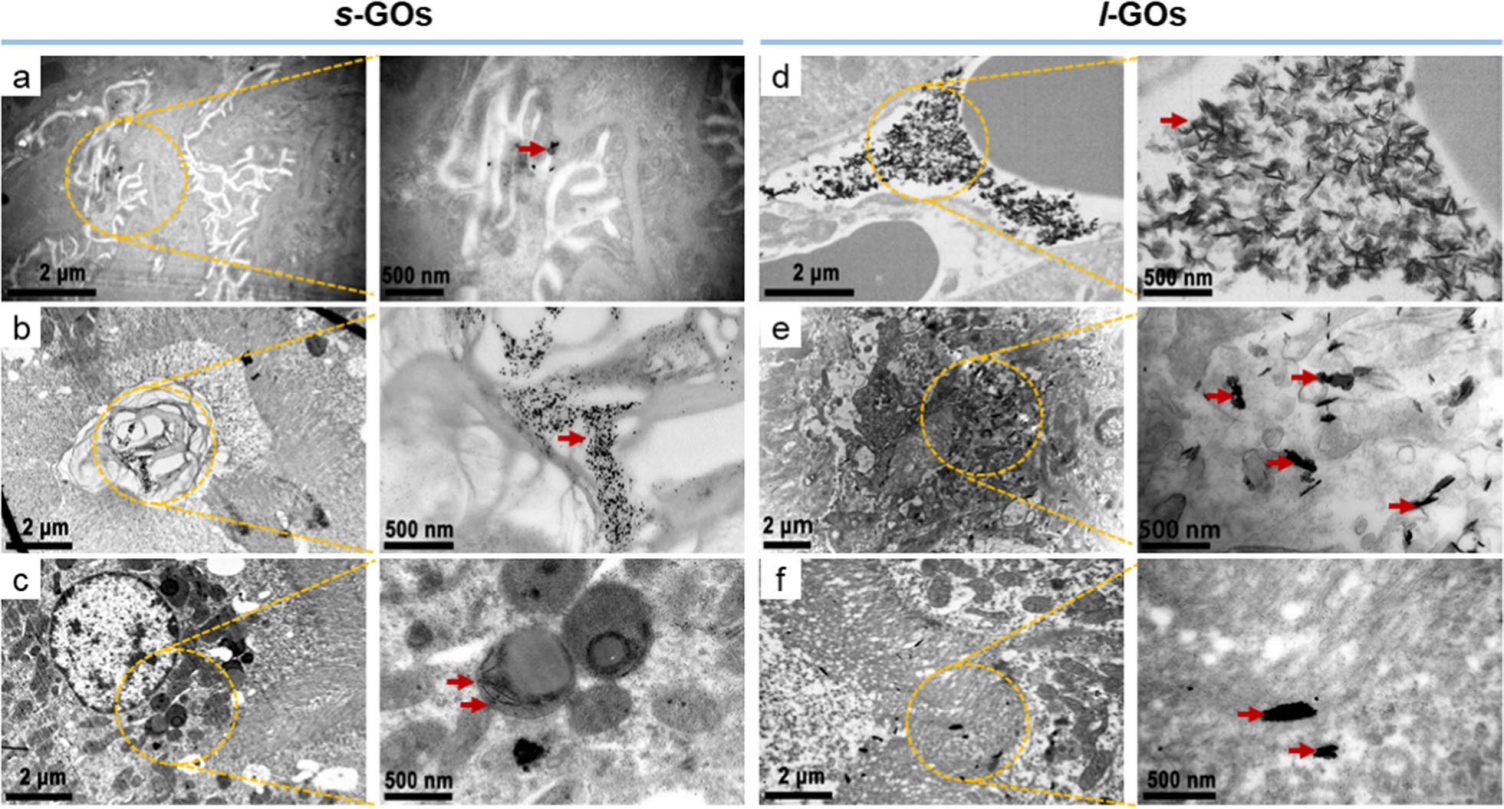
Ultrastructure imaging of the glomerulus and proximal tubule by TEM at 4 h after i.v. injection of *s*-GOs and *l*-GOs. *s*-GOs were shown depositing in podocyte foot process (**a**), the renal tubular space (**b**) and the cytosol of renal tubule epithelial cell (**c**). *l*-GOs deposited in peritubular capillary (**d**), tubular interstitium (**e**) and tubular lumen (**f**). Clusters of GOs were indicated by red arrows

The renal clearance depends on processes of glomerular filtration, tubular secretion and reabsorption of which the tubular processes take place predominantly in the proximal tubule. We then used TEM imaging to explore the ultrastructural changes of glomerulus and proximal tubule after *s*-GO and *l*-GO administrations. In the *s*-GO treated mice, a small amount of GOs were found accumulating in the podocyte foot process (which line the epithelial side of the GBM), the renal tubular space and the cytosol of renal tubular epithelial cell at 4 h post-injection (Fig. 3, Additional file 1: Fig. S9), indicating that the *s*-GOs entered into the glomerular capsule and parts of them passed from the glomerulus into the renal tubule or in the meanwhile had the processes of tubular secretion and reabsorption. While, in the *l*-GO treated mice, a considerable amount of GOs were observed to accumulate in the peritubular capillary, tubular interstitium and tubular lumen, suggesting the process of proximal tubular secretion of *l*-GOs.

**Fig. 4:**
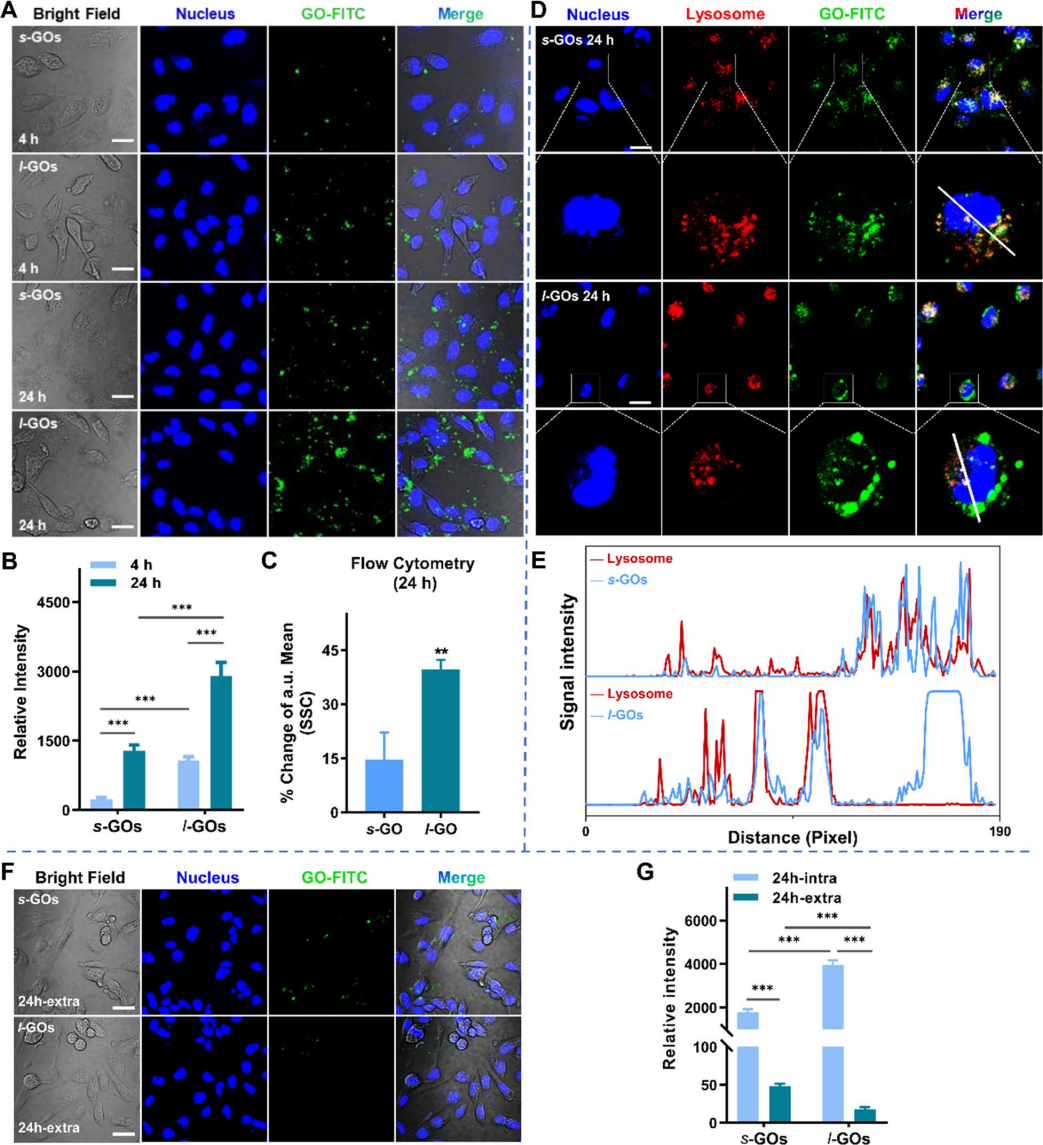
Confocal microscopy showing cellular uptake, localization and exocytosis of *s*-GOs and *l*-GOs in HK-2 cells. **a** Images of cellular uptake of FITC labelled (green) *s*-GOs and *l*-GOs after 4 and 24 h coculture. The nucleus was stained with DAPI (blue). **b** Quantitatively analysis of uptake of *s*-GOs and *l*-GOs in HK-2 cells. **c** Quantitative analysis of GO internalized in cells after 24 h treatment of *s*-GOs and *l*-GOs compared to untreated group by flow cytometry **d** Subcellular colocalization analysis of *s*-GOs-FITC/*l*-GOs-FITC and LysoTracker Red in HK-2 cells after 24 h coculture. The nucleus was stained with DAPI (blue). The second line images of each GO-treated group show higher magnification of a cell. **e** Profiles of fluorescence signal intensity along the straight line (white) crossing the nucleus and lysosome of a representative *s*-GO/*l*-GO treated cell. **f** Exocytosis of *s*-GOs and *l*-GOs from HK-2 cells after GO-treated cells cultured in GO-free medium for 24 h. **g** Quantitatively analysis of *s*-GO or *l*-GO exocytosis from HK-2 cells. Scale Bar: 20 μm; intra: intracellular; extra: extracellular. ***p < 0.001

We subsequently track the uptake and efflux of FITC-labeled GOs (GO-FITC) in HK-2 renal proximal tubular cells using confocal fluorescence microscopy. As shown in Fig. 4a, the fluorescence intensities of *s*-GOs/FITC and *l*-GOs/FITC (green) in HK-2 cells increased with the incubation time. The quantitative analysis indicates that the uptake of *l*-GOs in HK-2 cells is about fourfold and twofold higher than that of *s*-GOs after 4 h and 24 h coculture, respectively (Fig. 4b). Further, flow cytometry analysis also demonstrated that HK-2 cells showed about twofold higher intracellular uptake of *l*-GOs/FITC than that of *s*-GOs/FITC counterparts at 24 h coculture (Fig. 4c). We found that once GOs entered into the cells, a large portion of *s*-GOs were trafficked to lysosome, in contrast, a portion of *l*-GOs escaped the lysosome capture (Fig. 4d, f). The higher portion of *l*-GOs in the lysosome of HK-2 cells compared to that of the smaller counterparts is similar with many previous results that the smaller sized nanoparticles are more prone to translocate into the lysosome than the larger sized ones [31, 32]. The efflux of *s*-GOs and *l*-GOs from HK-2 cells was further analyzed. As shown in Fig. 4f, after GOs 24-h coculture with HK-2 cells, a few amounts of *s*-GO fluorescence signals (green) were observed within the cells after 24 h exocytosis, comparatively, *l*-GO fluorescence were hardly found, suggesting the efflux of *s*-GOs and *l*-GOs from HK-2 cells (Fig. 4g). The quantitative analysis shows that the HK-2 uptake and exocytosis of *l*-GOs were much more than that of *s*-GOs. The CCK-8 assay shows that 1 ~ 200 μg/mL of *s*-GO or *l*-GO treatments induced obvious dose-dependent decrease of cell viability in HK-2 cells when the doses is higher than 50 μg/mL (Additional file 1: Fig. S10). Comparatively, the *l*-GO treatment led more severe cytotoxicity than the *s*-GO did because significantly higher inhibition of cell growth was induced by *l*-GOs under the corresponding dose than by *s*-GOs.

### Insight into the expression and regulation of drug transporters in renal tubular uptake and secretion of s-GOs and l-GOs

**Fig. 5:**
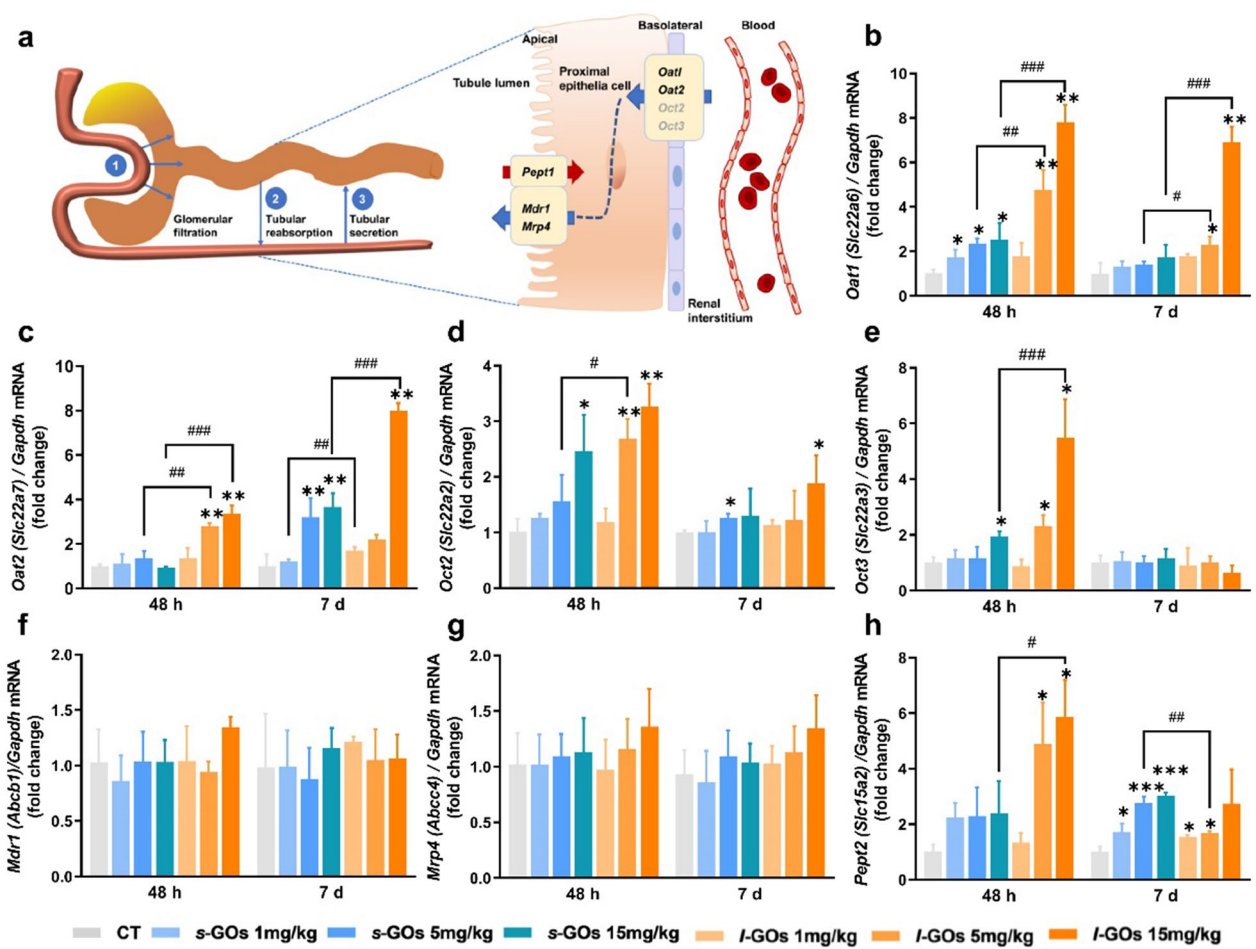
**a** A schematic diagram of the three concurrent processes of renal clearance: glomerular filtration, tubular secretion and tubular reabsorption, and the major renal tubular drug transporters involved in the uptake and secretion of *s*-GOs and *l*-GOs. **b** The mRNA expression levels **b**–**h** of renal tubular drug transporters, including organic anion transporter 1 (*Oat1*/*Slc22a6*) and 2 (*Oat2*/*Slc22a7*), organic cation transporter 2 (*Oct2*/*Slc22a2*) and 3 (*Oct3*/*Slc22a3*), ABC-transporters *Mdr1* (*Pgp/Abcb1*) and *Mrp4/Abcc4,* and peptide transporter 2 (*Pept2*/*Slc15a2*) at 48 h and day 7 after i.v. injection of *s*-GOs and *l*-GOs at doses of 1, 5, 15 mg/kg bw (n = 3). *p < 0.05, **p < 0.01 and ***p < 0.001 indicate the statistical difference between treated group and the controls. ^#^*p* < 0.05, ^##^*p* < 0.01 and ^###^*p* < 0.001 indicate the statistical difference between *s*-GO and *l*-GO treated groups

Approximately 80% renal blood flows through the efferent arterioles to the peritubular capillaries, where uptake from the plasma and excretion of solutes and medications, or vice versa, reabsorption from urine is mediated by transporters expressed on the basolateral and apical membrane of proximal tubules (Fig. 5a) [33]. The organic anion transporters (OATs) and organic cation transporters (OCTs) of the solute carrier 22 (SLC22) family play a major role in the handling of common drugs and toxins [33, 34].

The transporter mRNA expression detection shows that the basolateral transporters of *Oat1*/*Slc22a6*, *Oat2*/*Slc22a7*, *Oct2*/*Slc22a2* and *Oct3*/*Slc22a3* present dose-dependent elevation in the kidneys of both *s*-GO and *l*-GO treated mice compared to those in the control mice at 48 h post-injection (Fig. 5b–h). In particular, the *Oat1* and *Oat2* levels in the medium (5 mg/kg) and high dose (15 mg/kg) *l*-GO treated mice increased 2- to eightfold as compared with the controls and most of the levels were also statistically higher (*p* < 0.05, *p* < 0.001) than those in the corresponding dose of *s*-GO treated mice, strongly suggesting that these anion organic transporters facilitate the uptake of GOs in proximal tubular cells. Comparatively, the highest elevation of *Oct2* and *Oct3* was only threefold compared with the controls, much lower than the levels of *Oat1* and *Oat2*. The apically-expressed ABC-transporters: *Mdr1*/*Pgp* (*Abcb1*) and *Mrp4*/*Abcc4*, which are the key regulators for metabolism and efflux drugs [35], and peptide transporter 2 (*Pept2*/*Slc15a2*), which contributes to the tubular reabsorption, were detected. However, no obvious alteration of the mRNA levels of *Mdr1*/*Abcb1* and *Mrp4*/*Abcc4* were found in *s*-GO and *l*-GO treated mice. The *Pept2*/*Slc15a2* mRNA presented dose-dependent elevation in *s*-GO and *l*-GO treated mice at 48 h and day 7 post-injection. The highest level was found approximately sixfold elevation in the high dose of *l*-GO treated mice at 48 h and threefold elevation in the *s*-GO treated mice at day 7 post-injection.

### Kidney injuries during s-GO and l-GO renal excretion

**Fig. 6:**
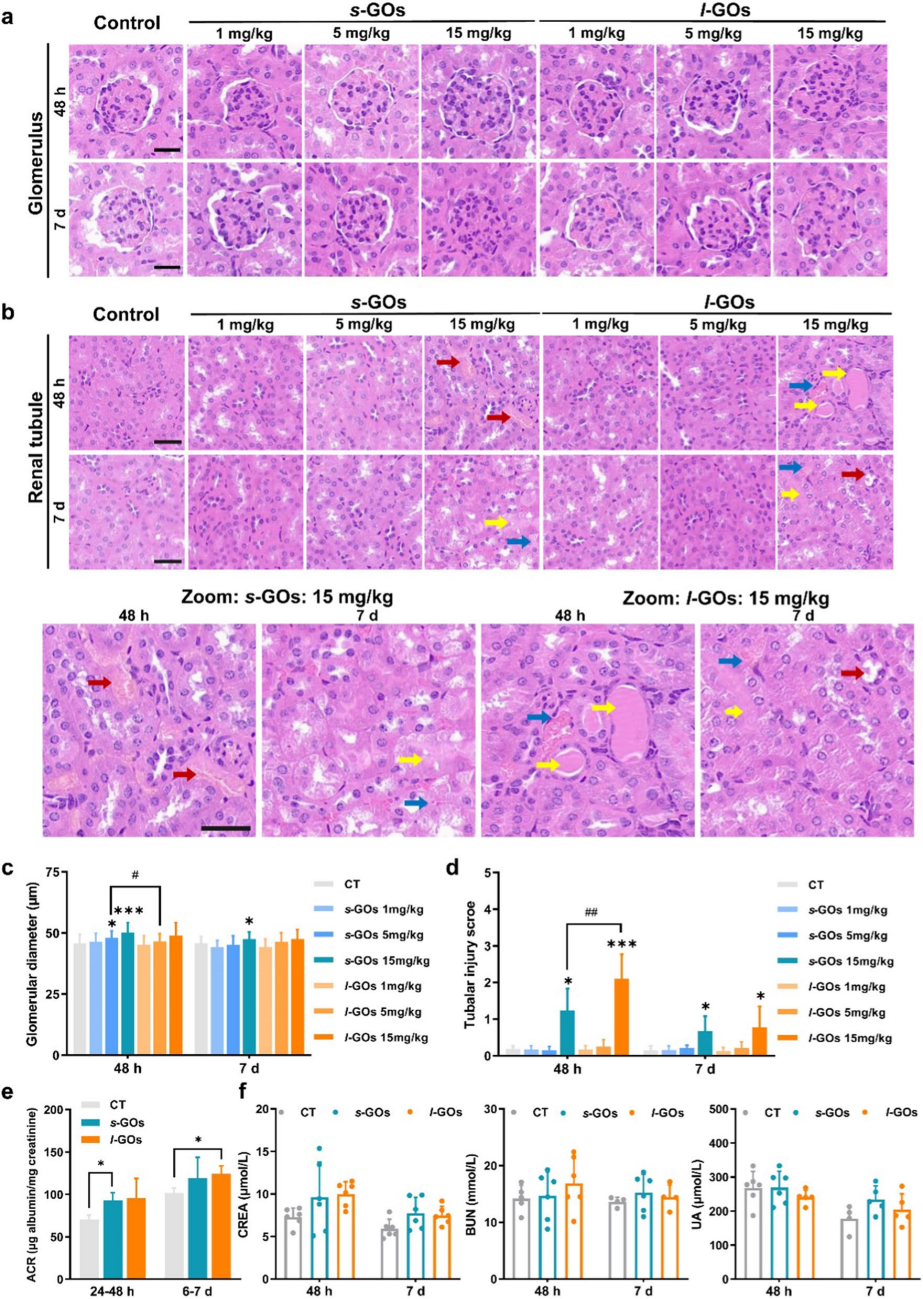
Histopathological examination and changes of biomarkers of renal function in the *s*-GO and *l*-GO administrated mice at 48 h and day 7 post-injection. **a** Histopathological alterations in glomerulus (n = 3), magnification: × 20. **b** Histopathological alterations in renal tubule (n = 3), magnification: × 20 (upper panel), × 40 (lower panel). The red arrows indicate necrosis of renal tubular epithelial cells; the yellow arrows indicate cast formation and the blue arrows indicate the tubular dilatation. **c** Statistical analysis the changes of glomerular diameter in *s*-GO and *l*-GO administrated mice. **d** Scoring of tubular injury. Tubular injury was defined as necrosis, loss of brush border, cast formation and tubular dilatation. Six cortical fields of view (× 200 magnification) were randomly selected and the tubular injury was evaluated according to the following scoring system: 0 = no tubular injury; 1 ≤ 10% tubules injured; 2 = 11–25% tubules injured; 3 = 26–50% tubules injured; 4 = 51–75% tubules injured; 5 > 75% tubules injured. **e** Measurement of 24-h urinary albumin-creatinine ratio (ACR) after injected of 15 mg/kg bw *s*-GOs and *l*-GOs to mice (n = 3). **f** Serum levels of the biomarkers of renal function after i.v. injection of 15 mg/kg *s*-GOs and *l*-GOs to mice (n = 5). CREA: creatinine; BUN: urine nitrogen; UA: uric acid. Scale Bar: 40 μm; GBM: glomerular basement membrane. *p < 0.05 and ***p < 0.001 indicate the statistical difference between treated group and the controls. ^#^*p* < 0.05 and ^##^*p* < 0.01 indicate the statistical difference between *s*-GO and *l*-GO treated group

Since the experimental evidence shows that the *s*-GO and *l*-GO could be excreted by the way of kidneys, then the most important clinical question is whether the processes could induce renal injury. We performed histological examinations of the kidney at 48 h and day 7 after i.v. injection of GOs in mice (Fig. 6a, b). The glomerular diameter was observed increase along with injected dose in the *s*-GO and *l*-GO treated mice (Fig. 6c). No obvious pathological changes were observed in the renal tubule of 1 or 5 mg/kg *s*-GOs and *l*-GOs injected mice. The obvious tubular injuries including necrosis of renal tubular epithelial cells, cast formation and tubular dilatation were only observed in the high dose (15 mg/kg) of *s*-GOs and *l*-GOs administrated mice (Fig. 6d), meanwhile, significant increase of glomerular diameters was observed induced by the high dose of 15 mg/kg *s*-GO and *l*-GO treatment (Fig. 6c).

To assess the potential implications of renal clearance of GOs on GFB and proximal tubules, we measured the albumin-creatinine ratio (ACR) in 24-h urine samples. The ACR in both *s*-GO and *l*-GO urine samples present dose-dependent increase at two 24-h (24–48 h and day 6–7 post-injection) excretion (Additional file 1: Fig. S11), and statistical differences were found in the high dose of *s*-GO (24–48 h post-injection) and *l*-GO (day 6–7 post-injection) treated mouse urine (Fig. 6e). In addition, the serum biomarkers of renal function, including CREA, BUN and UA, presented no obvious changes in the *s*-GO and *l*-GO treated mice as compared to the control group (Fig. 6f).

**Fig. 7:**
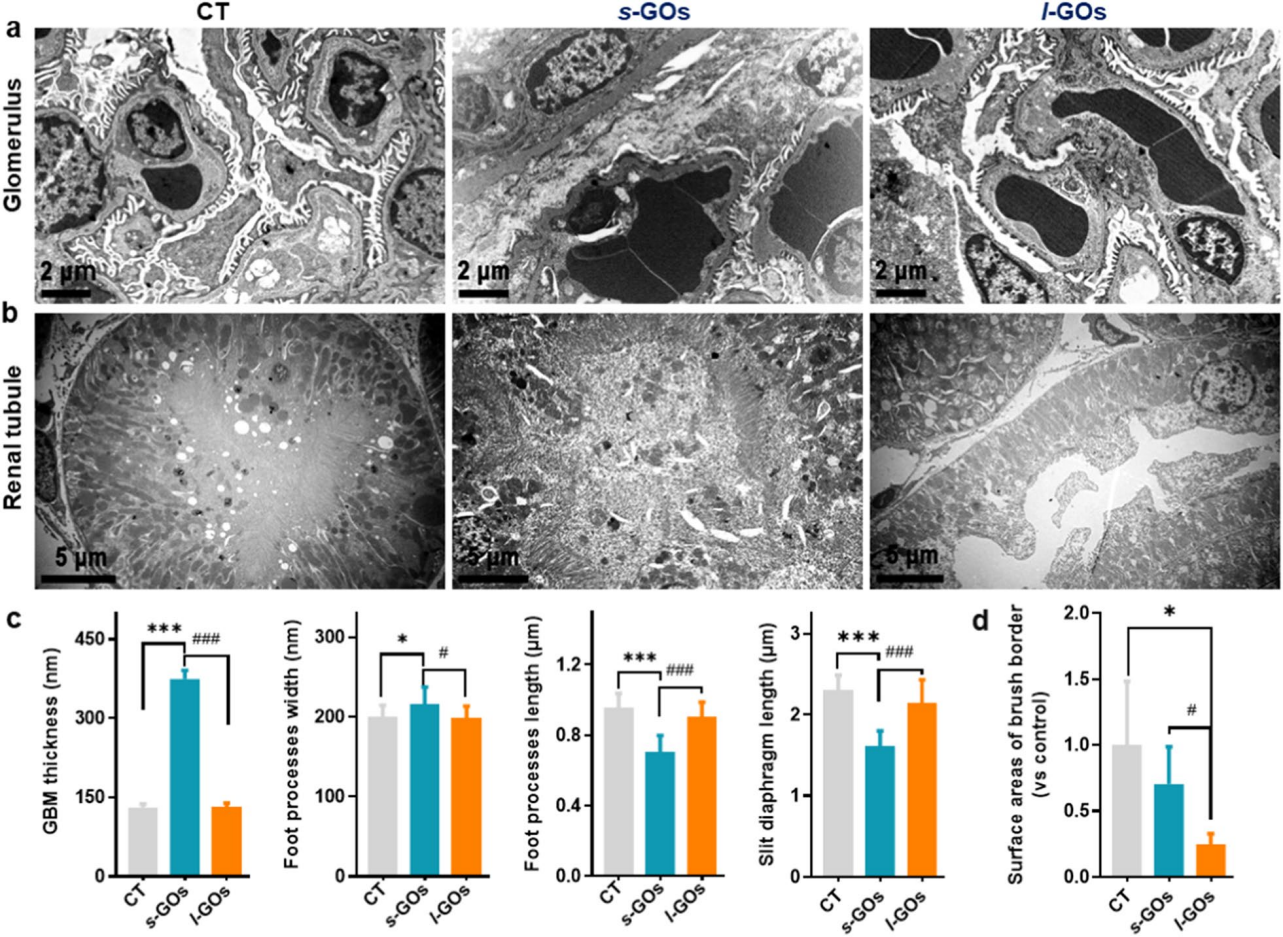
TEM images of kidney ultrastructure changes after i.v. injection of 15 mg/kg *s*-GOs and *l*-GOs at 48 h post-injection (n = 3). Ultrastructural changes of the glomerulus (**a**) and renal tubular (**b**). **c** Statistical measures of GBM thickness; podocyte foot process width; foot process length and slit diaphragm perimeter. **d** Statistical measures of renal tubule brush border loss. GBM: glomerular basement membrane. *p < 0.05 and ***p < 0.001 indicate the statistical difference between treated group and the controls. ^#^*p* < 0.05 and ^###^*p* < 0.001 indicate the statistical difference between *s*-GO and *l*-GO treated group

Observations of the ultrastructure of the kidney by TEM imaging further showed that the high dose (15 mg/kg) of *s*-GO treatment induced obvious impact on the structure of the GFB, including the increase of GBM thickness and podocyte foot process width, the decrease of foot process length and podocyte slit size after 48 h of injection (Fig. 7a, c). The podocyte foot processes appeared to be diffusely effaced in the *s*-GO mice (Fig. 7a). While, the 15 mg/kg of *l*-GO treatment caused visible tubular injury, such as renal vasodilation in the renal interstitium, renal tubule brush border loss, intratubular casts appearance, and epithelial cell necrosis (Fig. 7b, d).

To predict the severity of the kidney injury, we determined the mRNA expression levels of two biomarkers of acute kidney injury (AKI): kidney injury molecule-1 (KIM1) and neutrophil gelatinase-associated lipocalin (NGAL), and several inflammatory cytokines including chemokine (C–C motif) ligand 2 (*Ccl2*, also known as monocyte chemotactic protein-1: MCP-1), tumor necrosis factor alpha (*Tnfα*), interleukin-1 (*IL1β*) and interleukin-6 (*IL6)* in the kidneys (Additional file 1: Fig. S12a, b). The mRNA levels for KIM1 and NGAL did not show statistic upregulation even after high dose of 15 mg/kg *s*-GO and *l*-GO treatments. However, statistical elevation that more than 1.8- to tenfold increase of the expression levels of *Ccl2/Mcp1* and *Tnfα* in *s*-GO/*l*-GO kidney samples were found at 48 h and day 7 post-injection. No statistical elevation of mRNA for *IL1β* and *IL6* were detected.

## Discussion

Renal excretion including glomerular filtration, tubular secretion and reabsorption is the major route of elimination from the body for most drugs including nanomaterial-based medications. It is well known that NPs only with HD less than 6–8 nm can be effectively excreted in urine via glomerular filtration due to the size restriction of GFB which consists of endothelial fenestrae (70–90 nm), GBM (a mesh structure with 2–8 nm pores) and the epithelial filtration slits between podocyte processes (4–11 nm) [36]. Actually, for drug elimination, the proximal tubular secretion presents the primary kidney mechanism for rapidly eliminating the most endogenous solutes and hundreds of prescribed drugs from the circulation, including protein-bound molecules that cannot readily cross the GFB [34]. However, the renal tubular secretion of NMs is neglected and still seldom known to date. In this study, by using in vivo fluorescence imaging, LA-ICP-MS elemental imaging and confocal Raman spectroscopy, we firstly demonstrated efficient renal clearance of GOs and identified GO excretion in urine with the structure similar to its original. The kidney cortex disposition of GOs was demonstrated by in situ confocal Raman microscopy, TEM, LA-ICP-MS and FTIR imaging techniques. Importantly, we revealed that the pathway of GO renal clearance depends on its lateral dimension, *i.e.*, the small lateral sized *s*-GOs were excreted in urine by combination pathways of glomerular filtration and tubular secretion, while the large lateral sized *l*-GOs was mainly through tubular secretion. Correspondingly, the renal clearance rate of *l*-GOs was much faster than that of *s*-GOs.

Though there is a size restriction of NPs for glomerular filtration, if the shapes of NMs changed from zero-dimensional NPs to one-dimensional nanowires or two-dimensional nanosheets, the NM-kidney interactions would be much more complicated. For example, efficient urinary elimination of single walled and multiwalled carbon nanotubes with a diameter of 0.8–1.2 nm and lengths over 100 nm was observed [37]. In our previous work, we demonstrated that the thin and well-dispersed REE (La and Ce) labelled GO-PVP (polyvinylpyrrolidone) were excreted in urine from the kidney, and suggest a passive mechanism that the flexible GOs could reconfigure their morphology into filamentous shapes by rolling or folding for facilitating renal excretion, in addition, the negatively-charged glycocalyx covering on the luminal surface of the glomerular capillaries would also promote the negatively charged GO filtration [22]. Similar mechanism has been revealed in many other previous works as well [20, 21], and could explain the glomerular filtration of some *s*-GOs in this work. However, more importantly, we revealed that the active tubular secretion of GOs, especially for the large lateral dimensional *l*-GOs, is the primary route for GO renal elimination. We found that in *l*-GO administrated mice, lots of GOs deposited in the peritubular capillary, tubular interstitium and tubular lumen, suggesting the tubular secretion of *l*-GOs. Moreover, by using confocal fluorescence microscopy, we demonstrated that the quantities of uptake and exocytosis of *l*-GOs by HK-2 cells (human renal proximal tubule epithelia cells) were much higher than that of *s*-GOs.

When the solutes and medications flow through the peritubular capillaries, they will enter the interstitium and are actively transported into the proximal tubular cells via basolateral transporters (*i.e*., “uptake/influx” transporters), subsequently are transported into the tubule lumen via apical transporters (*i.e.*, “efflux” transporters) and finally excrete into the urine, or vice versa to be reabsorbed from urine [33, 34, 38]. As the first step for organic anion (OA) renal excretion, the OAT1-3 are highly expressed on the basolateral membranes of proximal tubules and function as predominantly renal anion-exchanging antiporters to mediate the uptake of drugs from the blood into the renal tubular cells [34]. In the study, for the mechanism exploration, we revealed that the basolateral organic anion transporters *Oat1*/*Slc22a6* and *Oat2*/*Slc22a7* contributed to the active uptake of GOs in proximal tubular epithelial cells, especially for the *l*-GOs that the mRNA expression levels of *Oat1* and *Oat2* in the kidneys of *l*-GO treated mice were statistically higher than those in the *s*-GO treated ones. It is noteworthy that the OAT1-3 are in some degree of multi-specific organic anion transporters with numerous xenobiotics and endogenous substrate thus have been highlighted by regulatory agencies as key transporters (especially OAT1) involved in drug excretion and potential drug-drug interactions (DDI) [39]. The anion efflux into the urine is an energy-dependent process that is generally mediated by members of the ATP binding cassette transporter family, including MRP2 and 4, the ATP-dependent transporter P-gp and so forth [40]. Generally, SLC (“uptake/influx”) transporters are combined with ABC (“efflux”) transporters for transepithelial vectoral transport involving proximal tubular secretion and reabsorption [33, 35, 39]. However, no obvious alterations of *Mdr1* and *Mrp4* mRNA levels were found in the *s*-GO and *l*-GO treated mice, thus, imply the risk of long-term deposition of GOs after administration. The hypothesis has been confirmed by the following comprehensive histological assessment. Interestingly, the mRNA level of *Pept2*/*Slc15a2*, which is involved in reabsorption process, was found dose-dependent and statistical elevation in the kidneys of *s*-GO and *l*-GO treated mice, however, the reabsorption of GOs by the renal tubules remains to be fully explored.

Medications are a common cause of acute kidney injury (AKI), especially, the excretion pathway of drugs via the proximal tubules may further contribute to the injury [41]. Previous studies have revealed that significant amounts of GO filtration through the glomeruli did not induce obvious sign of acute nephrotoxicity or glomerular barrier dysfunction when the mice were i.v. injected with GO solution at 2.5 mg/kg [22] or even at 7.5 mg/kg dosage [21]. This investigation further revealed that the GO-induced kidney injury associates with their lateral-size dependent renal excretion pathways. According to the conventional renal pathology scoring system, we showed that the small lateral sized *s*-GOs induced dose-dependent histopathological changes on the glomerulus due to parts of them excretion via glomerular filtration; while the large lateral sized *l*-GOs induced more obvious tubular injuries because of that *l*-GOs predominately were excreted via tubular secretion. We performed comprehensive examination of the ultrastructural changes of the glomeruli and renal tubules. We found that the high dose of *l*-GO administration (15 mg/kg) induced obvious tubular injury, including necrosis of renal tubular epithelial cells, loss of brush border, cast formation and tubular dilatation, and these injuries were more severe than those in the control and *s*-GO treated mice. We performed detailed measurements of the changes of glomeruli and found the high dose of *s*-GO administration (15 mg/kg) induced the increase of glomerular diameter, GBM thickness and podocyte foot process width, and the decrease of foot process length and slit diaphragm perimeter. In accordance with the histopathological findings, all of these glomerular changes were more obvious in *s*-GO mice than in the control and *l*-GO treated mice. The high dose of *s*-GOs induced more obvious effect on glomerulus than *l*-GOs might be related with their different renal clearance pathway. *s*-GOs could be renal excreted mainly via the glomerular filtration, due to that the *s*-GOs keep better stability than *l*-GOs in the biological medium and they could resist the impacts of relatively higher concentrations of proteins and salts [42], while the *l*-GOs were predominately excreted via proximal tubular secretion. In addition, the more obvious effect on glomerulus by *s*-GOs might be related with their interaction with the renal different types of cells during glomerular filtration, such as renal glomerular endothelial cells and podocytes, which need further studies.

It is hypothesized that the histopathological changes might affect renal excretion which is relative to glomerular filtration rate (GFR). We measured the GFR by albuminuria (*i.e.*, albumin-creatinine ratio, ACR) and serum creatinine (sCr), of which the albuminuria is confirmed as predictive biomarkers of early renal function decline [43], while the elevation of sCr is used for identification and evaluation severity of AKI [41]. We showed significant elevation of ACR in the high dose of *s*-GO and *l*-GO treated mice, which is related to the histological changes of the glomerulus and renal tubules, indicating the early sign of kidney injury. To explore the molecular mechanism of the kidney injury induced by the GO administration, we extended the detection to mRNA expression levels of two AKI biomarkers: KIM1 and NGAL, and several important inflammatory cytokines, including *Ccl2* (also known as *Mcp1*), *Tnfα*, *IL1β* and *IL6*, in the high dose (15 mg/kg) of GO treated kidney tissue lysates. The KIM1 and NGAL mRNA are proved as the sensitive and specific markers of tubular injury [44, 45]. KIM1 is expressed on the proximal tubule apical membrane with injury [44], while NGAL is found specific expression in distal tubular segments of injured nephrons [45]. A slight but not significant increase of KIM1 and NGAL levels were measured, however, meanwhile, approximately 1.8- to 3.5-fold elevation of *Ccl2/Mcp1* and *Tnfα* were detected in the 48 h or day 7 post-injected *s*-GO and *l*-GO mouse kidneys. The results suggest the inflammation in kidney could be induced during the process of the high-dose GO renal clearance. Overall, the renal injury evaluation indicates the high dose of *l*-GO treatment induced more obvious renal injury than *s*-GO did. One main reason is that the robust clearance properties of tubular secretion suggest the risk of proximal tubule substantially accumulate of high concentration of toxins when the proximal tubule epithelial cells show higher uptake of a drug (in the study, *i.e.*, GOs) than efflux it into the tubule lumen. We also found that the *l*-GO treatments induced more obvious dose-dependent cytotoxicity in HK-2 cells than *s*-GO did. Furthermore, the tubular transport processes can be interfered, *e.g.*, interactions with drug transporters. However, GO interaction with apical/basolateral proximal tubule transporters is still not known yet.

Collectively, our research demonstrates that the proper dose administrated GOs can be renally excreted via glomerular filtration or proximal tubular secretion as hundreds of commonly prescribed drugs do. This fact is important for GO clinical applications because the ideal medication dosing strategy would be on the basis of the kidney mechanism of drug elimination. For most administered medications that are cleared by the kidneys, proximal tubular secretion is the predominant mechanism. Furthermore, exploring the effects of NM physicochemical properties such as surface charge and surface modification on their interactions with the specific renal system components are the underlying principle of kidney disease diagnosis and management.

## Conclusion

**Fig. 8:**
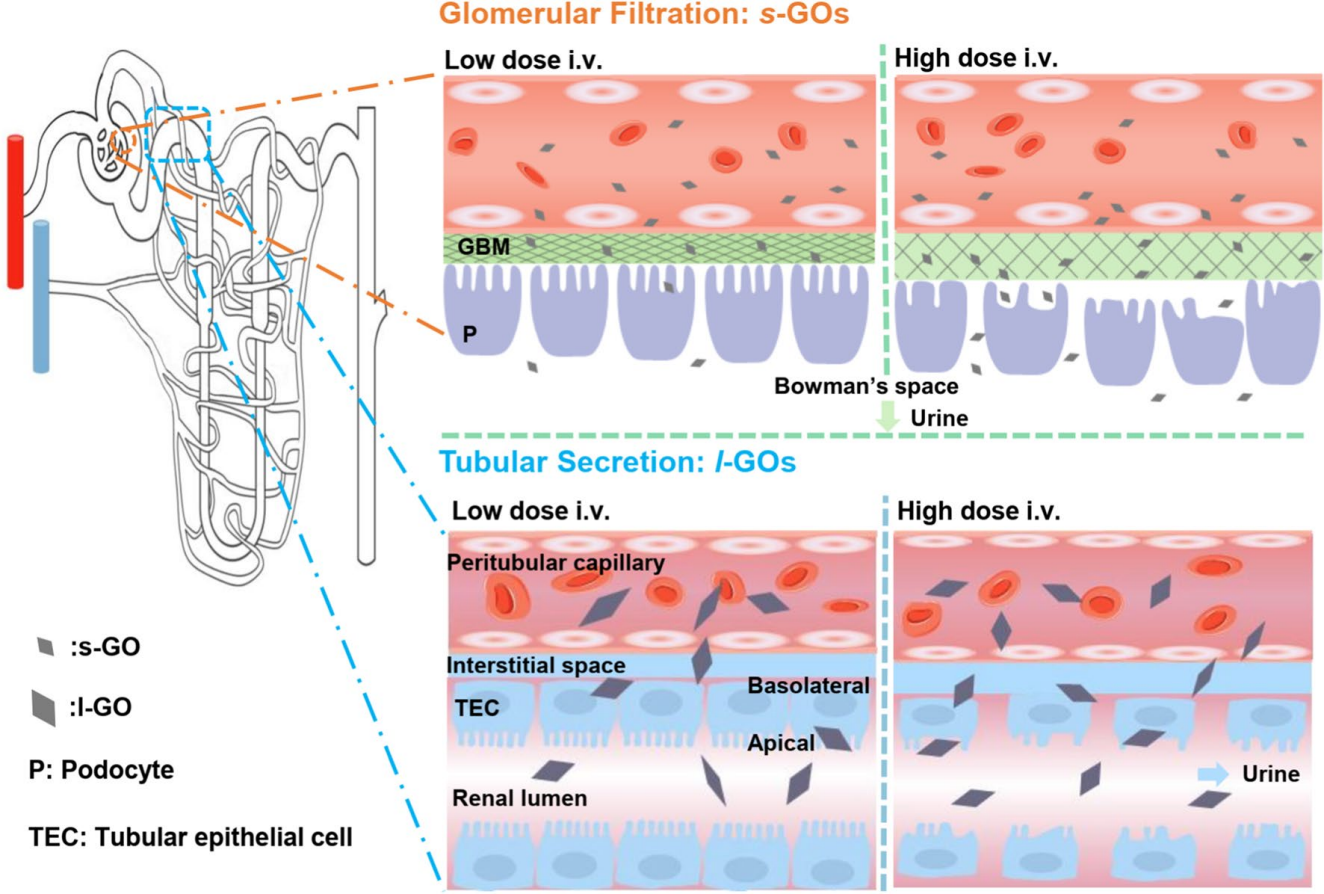
The lateral size affects renal clearance pathways of GOs and selectively induces potential risk of kidney injury. Graphene oxide with different lateral sizes showed different renal excretion pathways. The *s*-GOs can be excreted via glomerular filtration, while *l*-GO tends to be excreted into urine via tubular secretion. The basolateral/influx and apical/efflux transporters may contribute to the active uptake and secretion of GOs. There are dose dependent biological responses for glomerular filtration and tubular secretion of GOs. At a low dose administration, there are no obvious pathological changes in the kidneys in response to the glomerular filtration and tubular secretion. However, at a high dose administration, *s*-GO administration induced obvious glomerular changes, including increase of GBM thickness, effacement and diffuse fusion of foot processes, increase of foot process width and decrease of foot process length and slit diaphragm perimeter. The tubular secretion of large amounts of *l*-GOs caused obvious tubular injury, such as tubular dilatation, loss of the brush border on the surface of the renal proximal tubular epithelium, appearance of tubular casts, and epithelial cell necrosis

In summary, our study demonstrates that the thin, well-dispersed GOs can be eliminated from the body via the kidney, but the lateral dimension of GOs affects their renal clearance pathway. We highlighted that the small lateral sized *s*-GOs can be excreted via glomerular filtration, while the large lateral sized *l*-GOs would be predominately excreted via tubular secretion (Fig. 8). For the tubular secretion, the basolateral organic anion transporters Oat1 and Oat2 may contribute to the active uptake of GOs, especially for the *l*-GOs, in the proximal tubular epithelial cells. However, no obvious alterations of the efflux transporters such as *Mdr1* and *Mrp4* mRNA expression levels were observed, suggesting the possible of long-term accumulation of GOs after administration. The comprehensive histological assessments revealed that the renal clearance of GOs induced dose-dependent histopathological changes in glomeruli and renal tubules by *s*-GO and *l*-GO treatment, respectively. However, only the high dose (15 mg/kg) of GO administration affected the GFR of albuminuria, and meanwhile induced 1.8- to 3.5-fold increase of inflammatory cytokines *Ccl2/Mcp1* and *Tnfα* mRNA levels in the kidneys. The detection of the sensitive and cell specific biomarkers of KIM1 and NGAL for the tubular injury evaluation indicates slight increase of their mRNA levels in the *l*-GO treated mice kidneys. The histological and molecular results suggest the potential inflammation caused by *s*-GOs and *l*-GOs with high dose administration, especially by *l*-GOs in the renal proximal tubules. This study provided a principle of medication dosing strategy for GOs without impaired renal function in the biomedical applications.

## Supplementary Information

**Additional file 1:** **Table S1.** Sequences of primers used for RT-qPCR analysis in mouse kidney. **Figure S1. **Schematic diagram of Yb^3+^ labelled on DOTA-PEG functionalized GOs. **Figure S2. **(a) Dispersion stability of *s-*GOs and *l-*GOs in saline solution for 7 days. (b) Free Yb^3+^ concentration was analyzed by color reaction between xylenol orange (XO) and Yb^3+^. (c) The labelling stability was calculated as the ratio of the Yb mass on GOs (after subtracting free Yb^3+^) to the original total mass of Yb on GOs. **Figure S3.** In vivo fluorescence imaging of the biodistribution of s-GOs and l-GOs in mice and the clearance patterns from the body after i.v. injection of s-GOs/ICG and lGOs/ICG separately (n = 3). Dorsal side (a) and ventral side (b) imaged the in vivo clearance of s-GOs and l-GOs in mice. (c) Ex vivo fluorescence images of s-GO and lGO distribution in the 1) heart; 2) liver; 3) spleen; 4) lung and 5) kidneys. (d) Fluorescence intensity of s-GOs/ICG and l-GOs/ICG in the kidneys at 1, 4, 24, 48 h and day 7 post-injection. Fluorescence images were analyzed using Living Image 4.5.2. The unit of an average radiant efficiency: [p/s/cm2/sr]/[µw/cm2] is defined as the ratio of radiant flux per second in per area and per steradian to the excitation power density. **Figure S4. **Blood clearance curve in mice after i.v. injected of 5 mg/kg bw *s*-GOs and *l*GOs. n = 6. **Figure S5. **Biodistribution profiles of *s*-GOs and *l*-GOs after i.v. injection in mice. n = 6. **Figure S6. **(a) Typical FTIR spectra of graphene oxide (GO), PEGylated graphene oxide (GO-PEG), and mouse liver tissue. Band of 3439 cm^-1^ (O-H stretching) is assigned to GO; 1437 cm^-1^ (-CH_2_ stretching) and 1291 cm^-1^ (C-O-C stretching) are ascribed to PEG polymer. Synchrotron FTIR images (b) and quantitative analysis (c) of the mouse kidney after i.v. injection of *s*-GOs and *l*-GOs at 24 h i.v. post-injection. ** p < 0.001. **Figure S7. **Concentrations of ***s*-**GOs and ***l*-**GOs in stool samples after i.v. injection. **Figure S8. **Serum levels of the biomarkers of hepatic function after i.v. injection of 15 mg/kg *s*-GOs and *l*-GOs to mice (n = 5). **Figure S9. **Ultrastructure image of proximal tubule by TEM at 4 h after i.v. injection of *l*GOs. *l*-GOs deposited in peritubular capillary (a), tubular epithelial cytoplasm, (b) and tubular lumen (c). Clusters of GOs were indicated by red arrows. **Figure S10. **Cell viability assays of HK-2 cells following *s*-GO and *l*-GO treatments at 1, 10, 25, 50, 100, and 200 μg/mL doses for 24 h. ** p < 0.01 and *** p < 0.001 indicate the statistical difference between treated group and the controls. ### p < 0.001 indicate the statistical difference between *s*-GO and *l*-GO treated group. **Figure S11. **The measurements of 24-hour ACR (albumin to creatinine ratio) in urine after ***s*-**GO and ***l*-**GO injection to mice at doses of 1, 5, 15 mg/kg bw. **Figure S12. **(a) Schematic of the detection of biomarkers and inflammatory cytokines in the kidneys for evaluation of kidney injury after GOs administration. (b) The mRNA expression level changes (i-vi) of kidney injury molecule-1 (Kim1) and neutrophil gelatinase-associated lipocalin (Ngal), and the inflammatory cytokines: Ccl2/Mcp1, Tnfα, IL1β and IL6 in kidney lysates at 48 h and day 7 after i.v. injection of 15 mg/kg bw s-GOs and l-GOs to mice (n = 3).

## Data Availability

The data that support the findings of this study are available from the corresponding author upon reasonable request.
