# Supplementary material for: Renal clearance of graphene oxide: glomerular filtration or tubular secretion and selective kidney injury association with its lateral dimension
Source: J Nanobiotechnology. 2023 Feb 10;21:51. doi: 10.1186/s12951-023-01781-x (PMC9913007; doi:10.1186/s12951-023-01781-x)
Supplement: Supplementary file 1 — Additional file 1: Table S1. Sequences of primers used for RT-qPCR analysis in mouse kidney. Figure S1. Schematic diagram of Yb3+ labelled on DOTA-PEG functionalized GOs. Figure S2. (a) Dispersion stability of s-GOs and l-GOs in saline solution for 7 days. (b) Free Yb3+ concentration was analyzed by color reaction between xylenol orange (XO) and Yb3+. (c) The labelling stability was calculated as the ratio of the Yb mass on GOs (after subtracting free Yb3+) to the original total mass of Yb on GOs. Figure S3. In vivo fluorescence imaging of the biodistribution of s-GOs and l-GOs in mice and the clearance patterns from the body after i.v. injection of s-GOs/ICG and lGOs/ICG separately (n = 3). Dorsal side (a) and ventral side (b) imaged the in vivo clearance of s-GOs and l-GOs in mice. (c) Ex vivo fluorescence images of s-GO and lGO distribution in the 1) heart; 2) liver; 3) spleen; 4) lung and 5) kidneys. (d) Fluorescence intensity of s-GOs/ICG and l-GOs/ICG in the kidneys at 1, 4, 24, 48 h and day 7 post-injection. Fluorescence images were analyzed using Living Image 4.5.2. The unit of an average radiant efficiency: [p/s/cm2/sr]/[µw/cm2] is defined as the ratio of radiant flux per second in per area and per steradian to the excitation power density. Figure S4. Blood clearance curve in mice after i.v. injected of 5 mg/kg bw s-GOs and lGOs. n = 6. Figure S5. Biodistribution profiles of s-GOs and l-GOs after i.v. injection in mice. n = 6. Figure S6. (a) Typical FTIR spectra of graphene oxide (GO), PEGylated graphene oxide (GO-PEG), and mouse liver tissue. Band of 3439 cm-1 (O-H stretching) is assigned to GO; 1437 cm-1 (-CH2 stretching) and 1291 cm-1 (C-O-C stretching) are ascribed to PEG polymer. Synchrotron FTIR images (b) and quantitative analysis (c) of the mouse kidney after i.v. injection of s-GOs and l-GOs at 24 h i.v. post-injection. ** p < 0.001. Figure S7. Concentrations of s-GOs and l-GOs in stool samples after i.v. injection. Figure S8. Serum levels of the b [file 12951_2023_1781_MOESM1_ESM.pdf]

Supplementary Materials

Renal Clearance of Graphene Oxide: Glomerular Filtration or Tubular Secretion and Selective Kidney Injury Association with its Lateral Dimension

Wei Chen[1,2], Bing Wang[1,*], Shanshan Liang[1,2], Meng Wang[1], Lingna Zheng[1], Si Xu[1,3], Jiali Wang[1,3], Hao Fang[1,2], Pu Yang[1,2], Weiyue Feng[1,*]

[1] CAS Key Laboratory for Biomedical Effects of Nanomaterials and Nanosafety, Institute of High Energy Physics, Chinese Academy of Sciences, Beijing 100049, China

[2] University of Chinese Academy of Sciences, Beijing 100049, China

[3] School of Pharmacy in Yantai University, Yantai, 264005, China

*Corresponding author, Email address: Weiyue Feng, fengwy@ihep.ac.cn; Bing Wang, wangbing@ihep.ac.cn

Methods

Biodistribution analysis of GOs

The medium dose (5 mg/kg) of *s*-GOs and *l*-GOs were i.v. injected in CD-1 (ICR) mice and sacrificed at 4 h, 12 h, 24 h, 7 d and 28 d post-injection. The organic tissues of brain, heart, lung, liver, kidney, spleen, stomach, small intestine and testis were collected for organ distributions and clearance study.

Synchrotron radiation based FTIR Imaging analysis

The *s*-GO and *l*-GO suspensions were i.v. injected into CD-1 (ICR) mice at a dose of 5 mg/kg bw (n = 3). After 4 and 24 h of injection, the mice were sacrificed and perfused intracardially with 0.9% NaCl followed by 4% paraformaldehyde solution. The kidney tissues were collected and immediately frozen in liquid nitrogen. Frozen sections of 10 μm were placed on infrared transparent BaF2 slides and dried naturally [1].

The synchrotron radiation based FTIR imaging was performed at the BL01B beamline at Shanghai Synchrotron Radiation Facility (SSRF). The FTIR spectra were collected in transmission mode from 4000 to 800 cm^{-1} by a Nicolet 6700 Fourier transform infrared spectrometer with a Nicolet continuum infrared microscope.

Spectral imaging was obtained by raster scanning of the region of interest, *i.e.*, 20 × 20 μm^2 for the kidney and liver sections at a spectral resolution of 4 cm^{-1}. All the FTIR maps were processed using Omnic 9.0 (Thermo Fisher Scientific Inc.). The results were expressed as signal intensity per unit area.

Urinary albumin and creatinine clearance rate measurements

Mice were fed in metabolic cages to collect 24-hour urine at 24 ~ 48 h and day 6-7 post-injection for the measurement of urinary albumin and creatinine clearance rate (Ccr). The urinary albumin and Ccr levels were measured according to the protocols of the kits (Hengyuan Biotech, Shanghai, China). Briefly, urine was centrifuged with 1,000 *g* for 20 min at 4°C. The supernatant was collected, diluted and then added to a 96-well plate of which the enzyme labeled antibody was added previously. Then the mixture was incubated for 30 min at 37°C and followed by washing the plate with 0.05% Tween 20 contained PBS solution. After drying, a biotin solution labeled horseradish peroxidase was add and continuously incubated, then the plate washed with PBS (containing 0.05% Tween 20) and reacted with tetramethylbenzidine (TMB). The reaction was terminated with dilute sulfuric acid solution and the absorbance at 450 nm was measured. The Ccr was calculated according to the following formula:

Ccr = (urine creatinine [mg/dL] × urine volume [mL])/(serum creatinine [mg/dL] × 1440 [minutes])

Results

Synchrotron radiation FTIR imaging the transport of *s*-GOs and *l*-GOs in mice kidney

The SR-FTIR images have also been revealed the transport patterns of GOs as well as the surface PEG ligand in the kidney (Fig. S6). The typical FTIR spectra of GO, GO-PEG, and the GO-treated kidney slices are shown in Fig. S6. The IR spectrum show the typical absorption band of GO at ~ 3439 cm^{-1} (O-H stretching) and the characteristic absorption bands of PEG polymer at ~ 2963 cm^{-1} ($-CH_2/CH_3$ stretching) and ~ 1437 cm^{-1} ($-CH_2$ stretching) and ~ 1291 cm^{-1} (C-O-C stretching). Later, the absorption bands at ~ 1437 cm^{-1} and ~ 1291 cm^{-1} were used to identify the PEG polymer because the ~ 2963 cm^{-1} peak was disturbed by the signals from endogenous biological molecules.

The SR-FTIR images of the renal cortex at 24 h of GO post-injection are shown in Fig. S6b& S6c Comparing with the peak intensities of GO and PEG in the control and *s*-GO treated mice, the PEG band in the region of 1250 ~ 1360 cm^{-1} and at 1437 cm^{-1}, GO band in the region of 3400 ~ 3500 cm^{-1} in the *l*-GO treated mice significantly increased ($p < 0.001$), suggesting that the content of *l*-GOs in the renal cortex is significantly higher than those in *s*-GO treated and control mice.

References

1. Li X, Yu H, Wang B, Chen W, Zhu M, Liang S, et al. Multiscale Synchrotron-Based Imaging Analysis for the Transfer of PEGylated Gold Nanoparticles In Vivo. ACS Biomater. Sci. Eng. 2021;7:1462-1474.

Table S1. Sequences of primers used for RT-qPCR analysis in mouse kidney

Genes	Forward primers sequences (5'-3')	Reverse primers sequences (5'-3')
Gapdh	AATGGTGAAGGTCGGTGT	GTGGAGTCATACTGGAACATGTAG
Oat1	GCAGCCTATGCACCCAACTA	CTAGTGGCAAACCACAGCAT
Oat2	GCAGCCTCGGTCAACTACAT	CCAGCCACTCTAACTCCAACG
Oct2	GCCTTTACTGTTGGGCTCCT	GACTCCGGTATGCACCAGAAA
Oct3	GGGACTTATCGGAGGCAACC	CAGCCGAAAGAGCAGAAACG
Mdr1	TCCTTCTGCCTCTTTACCCT	GTCAATGCTTGGCTCGTTATCA
Mrp4	TCATTAGGCGGTTGTCCG	AGTTCTTTATCCCAGTACCGTTG
Pept2	AGAAGTCAACTCCGAAGCTC	TCCTTTTCCACTCCACTCACC
Kim1	CAGGAAGACCCACGACTATTTC	GTGTGTAGATGTTGGAGGAGTG
Ngal	CCACCACGGACTACAACCAG	AGCTCCTTGGTTCTTCCATACA
Ccl2	CACTCACCTGCTGCTACTCA	GCTTGGTGACAAAAACTACAGC
TNFα	CAGACCCTCACACTCAGATCATCT	CCTCCACTTGGTGGTTTGCTA
IL1β	TGCCACCTTTTGACAGTGATG	AAGGTCCACGGGAAAGACAC
IL6	TGGAAATGAGAAAAGAGTTGTGC	GAGAGCATTGGAAATTGGGGTAG

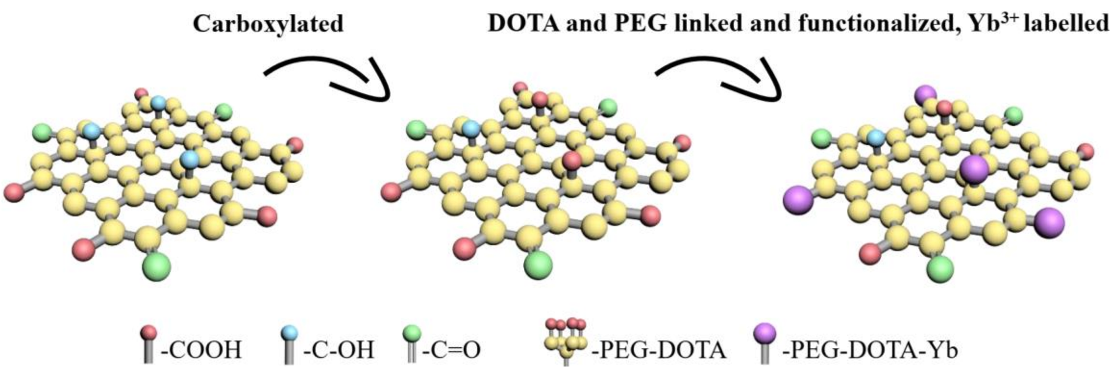

Fig. S1 Schematic diagram of Yb^{3+} labelled on DOTA-PEG functionalized GOs.

a

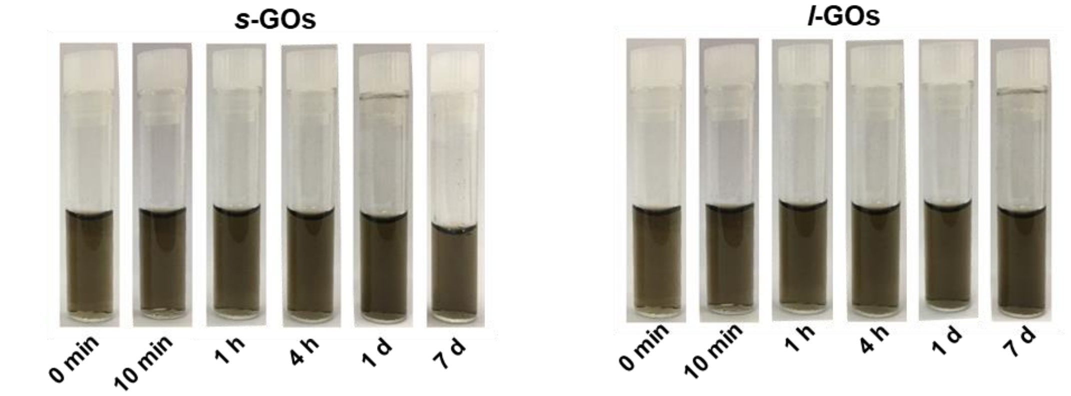

b

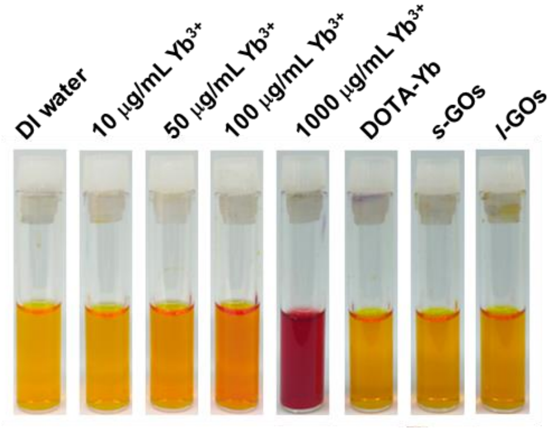

c

	s-GOs	*l*-GOs
Yb labelling stability (%)	99.2	99.6

Fig. S2 (a) Dispersion stability of *s*-GOs and *l*-GOs in saline solution for 7 days. (b) Free Yb^{3+} concentration was analyzed by color reaction between xylenol orange (XO) and Yb^{3+}. (c) The labelling stability was calculated as the ratio of the Yb mass on GOs (after subtracting free Yb^{3+}) to the original total mass of Yb on GOs.

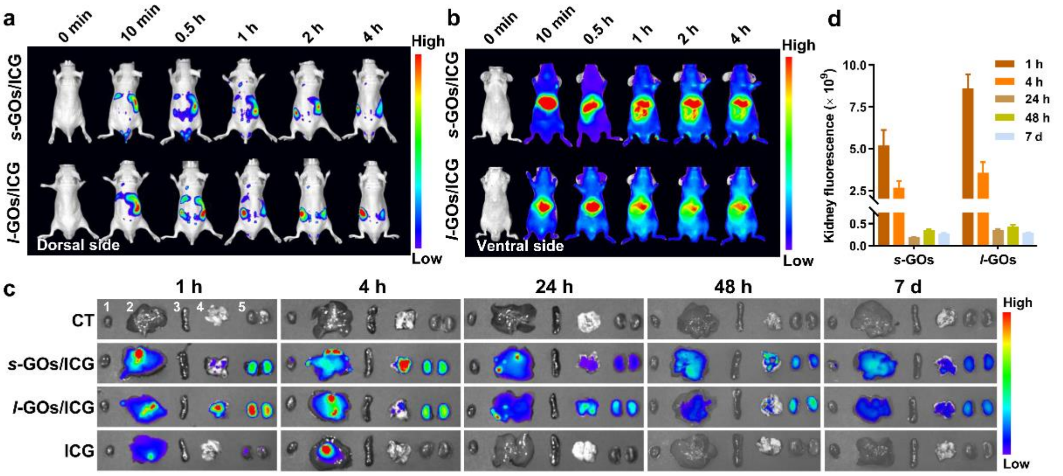

Fig. S3 In vivo fluorescence imaging of the biodistribution of s-GOs and l-GOs in mice and the clearance patterns from the body after i.v. injection of s-GOs/ICG and l-GOs/ICG separately (n = 3). Dorsal side (a) and ventral side (b) imaged the in vivo clearance of s-GOs and l-GOs in mice. (c) Ex vivo fluorescence images of s-GO and l-GO distribution in the 1) heart; 2) liver; 3) spleen; 4) lung and 5) kidneys. (d) Fluorescence intensity of s-GOs/ICG and l-GOs/ICG in the kidneys at 1, 4, 24, 48 h and day 7 post-injection. Fluorescence images were analyzed using Living Image 4.5.2. The unit of an average radiant efficiency: [p/s/cm2/sr]/[μw/cm2] is defined as the ratio of radiant flux per second in per area and per steradian to the excitation power density.

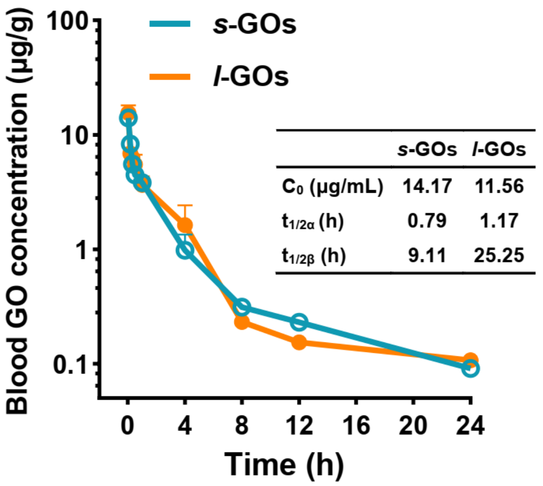

Fig. S4 Blood clearance curve in mice after i.v. injected of 5 mg/kg bw *s*-GOs and *l*-GOs. n = 6.

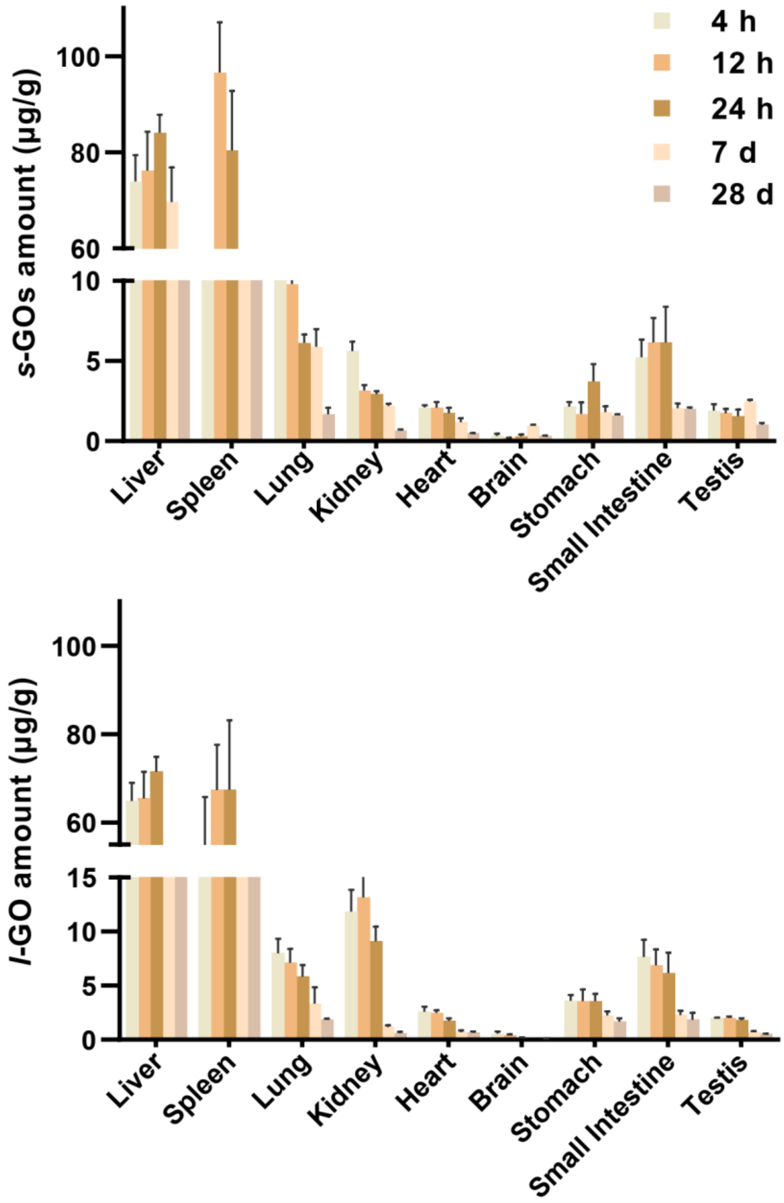

Fig. S5 Biodistribution profiles of *s*-GOs and *l*-GOs after i.v. injection in mice. n = 6.

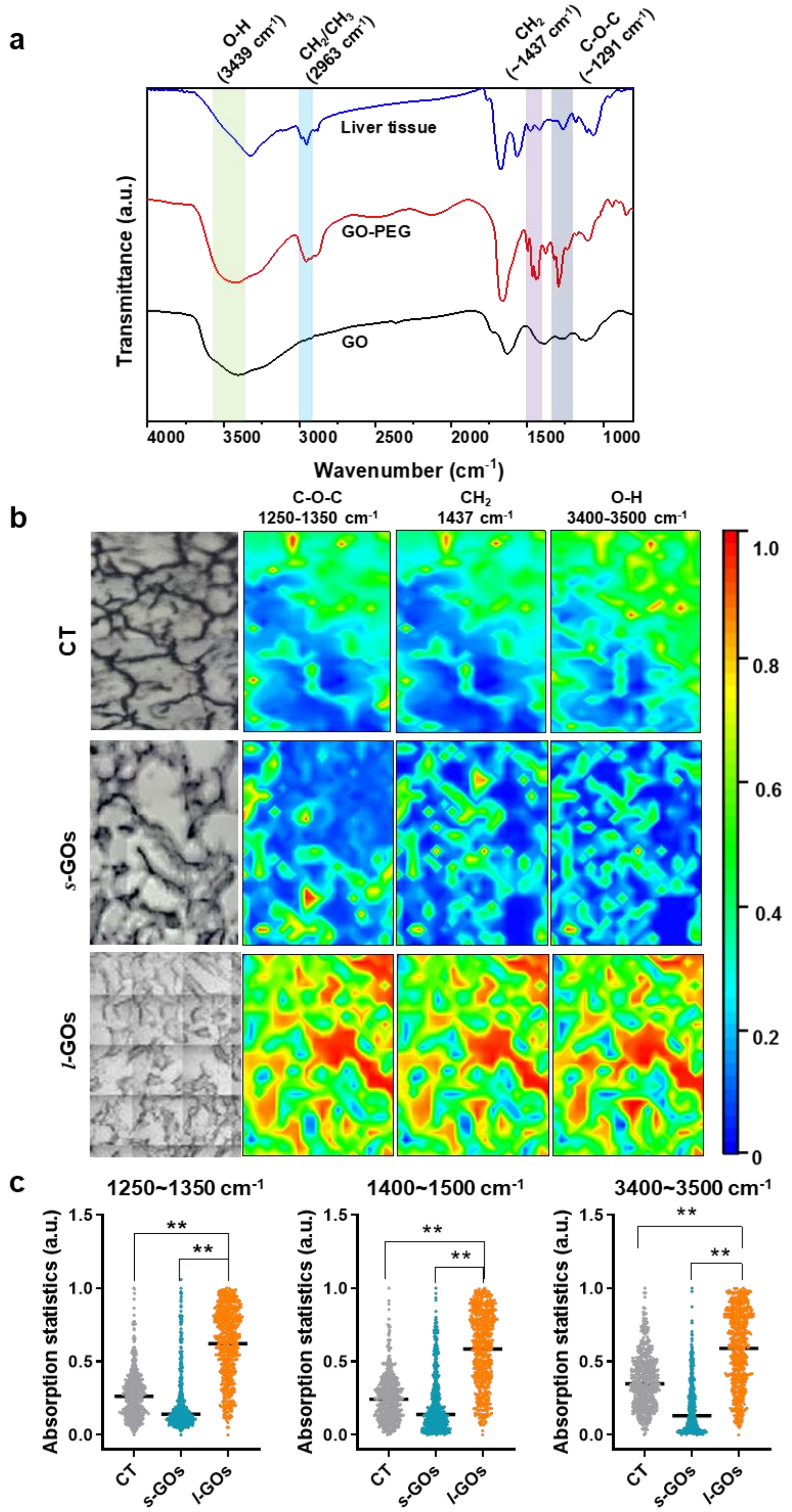

Fig. S6 (a) Typical FTIR spectra of graphene oxide (GO), PEGylated graphene oxide (GO-PEG), and mouse liver tissue. Band of 3439 cm^{-1} (O-H stretching) is assigned to GO; 1437 cm^{-1} (-CH_2 stretching) and 1291 cm^{-1} (C-O-C stretching) are ascribed to PEG polymer. Synchrotron FTIR images (b) and quantitative analysis (c) of the mouse kidney after i.v. injection of *s*-GOs and *l*-GOs at 24 h i.v. post-injection. ** $p < 0.001$.

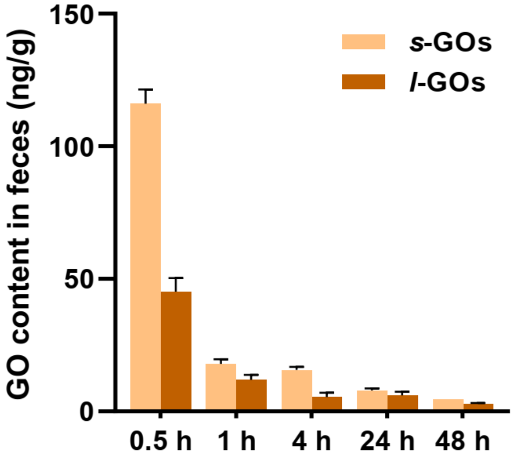

Fig. S7 Concentrations of ***s***-GOs and ***l***-GOs in stool samples after i.v. injection.

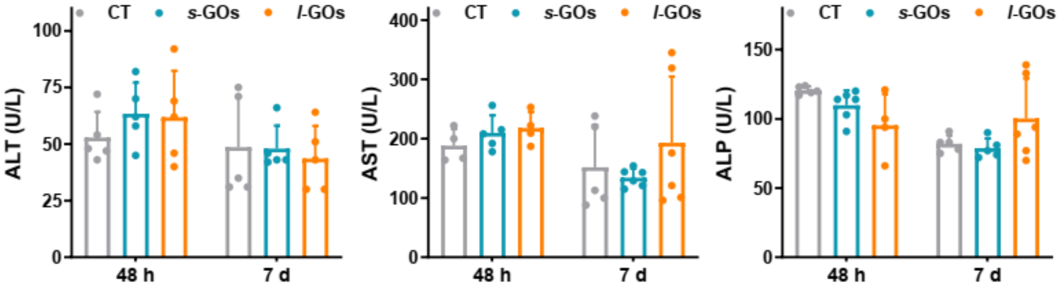

Fig. S8 Serum levels of the biomarkers of hepatic function after i.v. injection of 15 mg/kg *s*-GOs and *l*-GOs to mice (n = 5).

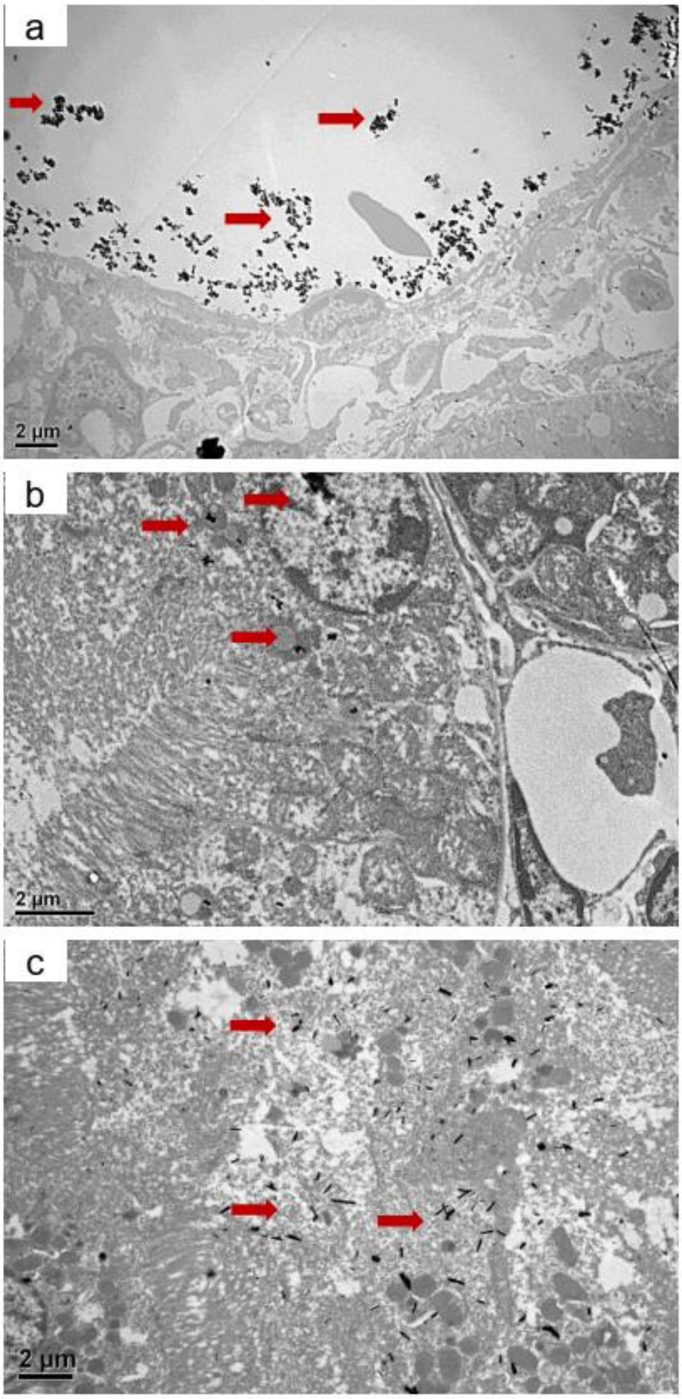

Fig. S9 Ultrastructure image of proximal tubule by TEM at 4 h after i.v. injection of *l*-GOs. *l*-GOs deposited in peritubular capillary (a), tubular epithelial cytoplasm, (b) and tubular lumen (c). Clusters of GOs were indicated by red arrows.

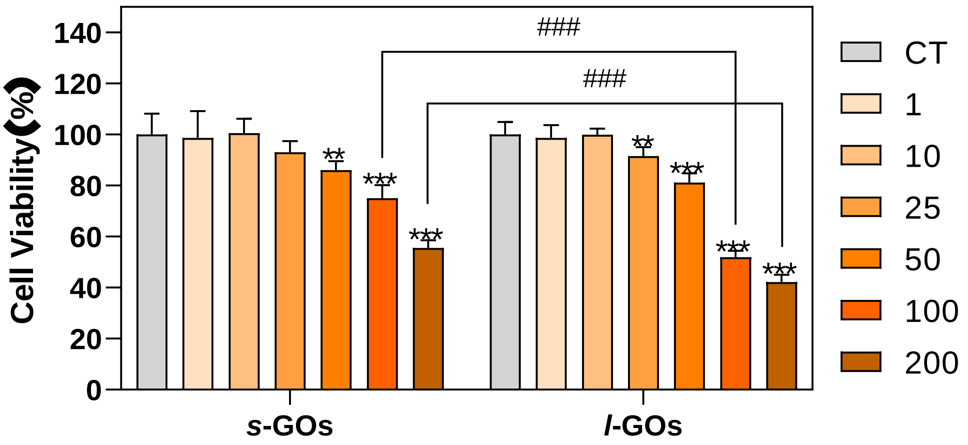

Fig. S10 Cell viability assays of HK-2 cells following *s*-GO and *l*-GO treatments at 1, 10, 25, 50, 100, and 200 μg/mL doses for 24 h. ** $p < 0.01$ and *** $p < 0.001$ indicate the statistical difference between treated group and the controls. ### $p < 0.001$ indicate the statistical difference between *s*-GO and *l*-GO treated group.

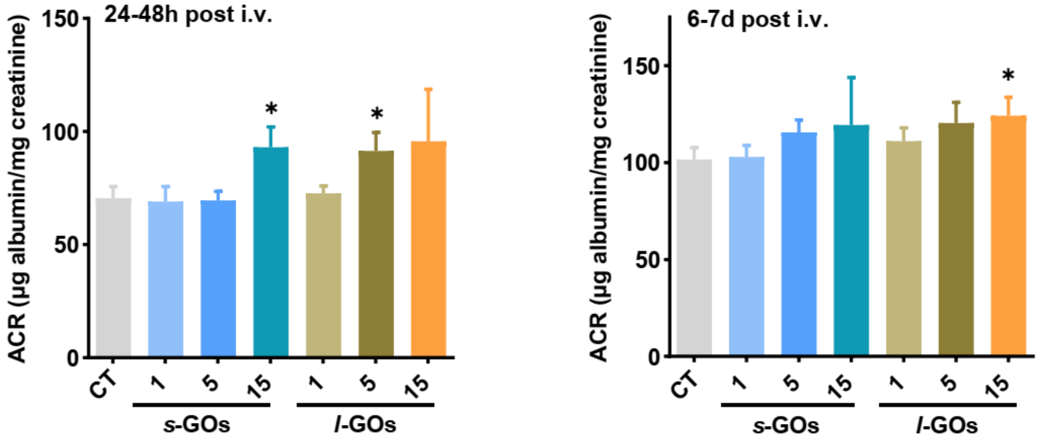

Fig. S11 The measurements of 24-hour ACR (albumin to creatinine ratio) in urine after ***s***-GO and ***l***-GO injection to mice at doses of 1, 5, 15 mg/kg bw.

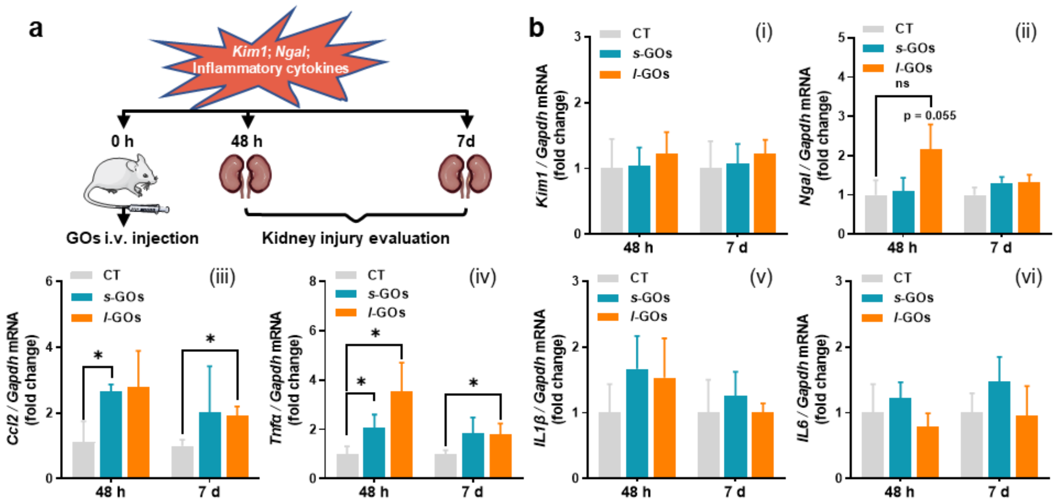

Fig. S12 (a) Schematic of the detection of biomarkers and inflammatory cytokines in the kidneys for evaluation of kidney injury after GOs administration. (b) The mRNA expression level changes (i-vi) of kidney injury molecule-1 (Kim1) and neutrophil gelatinase-associated lipocalin (Ngal), and the inflammatory cytokines: Ccl2/Mcp1, Tnfα, IL1β and IL6 in kidney lysates at 48 h and day 7 after i.v. injection of 15 mg/kg bw s-GOs and l-GOs to mice (n = 3).
